# Molecular Evidence of RNA Editing in *Bombyx* Chemosensory Protein Family

**DOI:** 10.1371/journal.pone.0086932

**Published:** 2014-02-13

**Authors:** Ning Xuan, Xun Bu, Yan Yan Liu, Xue Yang, Guo Xia Liu, Zhong Xue Fan, Yu Ping Bi, Lian Qun Yang, Qi Nian Lou, Balaji Rajashekar, Getter Leppik, Sergo Kasvandik, Jean-François Picimbon

**Affiliations:** 1 Biotechnology Research Center, Shandong Academy of Agricultural Sciences, Jinan, Shandong Province, China; 2 Shandong Silkworm Institute, Shandong Academy of Agricultural Sciences, Yantai, Shandong Province, China; 3 Institute of Computer Science, University of Tartu, Tartu, Tartumaa Province, Estonia; 4 Proteomics Core Facility, Institute of Technology, University of Tartu, Tartu, Tartumaa Province, Estonia; NIGMS, NIH, United States of America

## Abstract

Chemosensory proteins (CSPs) are small scavenger proteins that are mainly known as transporters of pheromone/odor molecules at the periphery of sensory neurons in the insect antennae and in the producing cells from the moth female pheromone gland.

Sequencing cDNAs of RNA encoding CSPs in the antennae, legs, head, pheromone gland and wings from five single individual adult females of the silkworm moth *Bombyx mori* showed that they differed from genomic sequences by subtle nucleotide replacement (RDD). Both intronless and intronic CSP genes expressed RDDs, although in different rates. Most interestingly, in our study the degree of RDDs in CSP genes were found to be tissue-specific. The proportion of CSP-RDDs was found to be significantly much higher in the pheromone gland. In addition, Western blot analysis of proteins in different tissues showed existence of multiple CSP protein variant chains particularly found in the pheromone gland. Peptide sequencing demonstrated the occurrence of a pleiad of protein variants for most of all BmorCSPs from the pheromone gland. Our findings show that RNA editing is an important feature in the expression of CSPs and that a high variety of RDDs is found to expand drastically thus altering the repertoire of CSP proteins in a tissue-specific manner.

## Introduction

DNA carries the genetic information of a cell, while the RNA carries the information encoded in DNA and produce proteins that perform important tasks for the cell function. It is assumed that the protein sequence that results from RNA translation perfectly reflects the sequence of DNA. It is also assumed that the transmission of information from DNA to RNA and RNA to protein is a very critical process and matter of strict surveillance within cells [1]. Thus, errors and edited RNA are supposed to be rare and if produced, they are immediately degraded. In modern organisms, multiple cellular proofreading and repair mechanisms function at all steps of the RNA biogenesis to ensure the fidelity and quality of transcription [2].

In humans, there are however thousands of places where transcribed RNA does not match DNA, rejecting the dogmatic ‘one-to-one’ relationship between DNA and RNA sequences [3]. These mismatches include errors in transcription and RNA-DNA differences (RDDs) that result from a specific mechanism called RNA editing [4]–[6]. RNA editing refers to all molecular processes in which a nucleotide mutation occurs changing the information content in the RNA molecule. Thus, from one single gene, the RNA editing mechanism can create proteins with slightly different functions. It is known for instance that humans (and insects) use RNA editing to create subtle differences in the properties of voltage-gated ion channels and neurotransmitter receptors [7]–[9], while viral RNA sequence variants can induce different virulence effects [10]. The widespread DNA-RNA differences due to RNA editing in transcriptome of organisms provide a yet unexplored aspect of genome expression.

A new family of chemosensory proteins (CSPs) has been described in most insect species [11]–[15]. The RNA encoding these proteins is particularly abundant in the antennae, legs and proboscis as well as in all other parts of the insect body. A function in olfaction and general corporal sensing has been proposed [16]–[17]. In the migratory locust *Locusta migratoria*, the phasmid *Carausius morosus* and the ant *Camponotus japonicus*, CSPs have been localized in the olfactory sensillae, the micro-organs responsible of odor detection in insects [18]–[20]. Such a high concentration of CSPs in olfactory units from the insect antennae is consistent with the notion that CSPs contribute to odor recognition [21]–[24].

However, this might not be the unique function of CSPs. In the cockroach *Periplaneta americana*, the CSP protein p10 (a molecule of 10 kDa; #AAB84283) is found to be highly expressed not only in the mature form of the antennae but also in regenerating legs. This suggests a dual developmental/chemosensory function for this protein [25], [26]. In moths, a few CSPs have been shown to be associated with developmental processing [27], [28]. A role of CSPs in insect development has been demonstrated in bees, where the lack of functional CSP gene in the honeybee *Apis mellifera* aborts normal head development [29].

Besides the antennae, legs and head, RNAs coding for CSPs have been found also in the pheromone gland of female moths. This suggests that these proteins have a pheromone-related function, e.g. storage of pheromones before release [30]–[33]. The structure of CSPs is well suited for a function as pheromone carriers. They are consistently made of six α-helices that fold into an open-air prism, well-suited to interact with long hydrocarbon “*pheromone-like*” aliphatic chains. The internal side of the α-helices from CSPs is covered with hydrophobic amino acid residues [34]–[37].

The problem comes out that the moth genome only holds twenty CSP genes similarly to butterflies [38], [39]. The number of CSPs in flies and bees (four-six) is found to be even lower [40], [41]. This poses unanswered question about how the number of CSP genes can cope to the detection of a million compounds, odorants, pheromones and other fatty acid derivatives. The 2D and 3D analysis of the binding pocket of the moth CSP has demonstrated the true capacity of the protein for key conformational changes to adopt multiple hydrophobic odorous ligands. Thus, moth CSPs can bind hydrophobic sensory ligands of all diverse shapes, sizes and composition [42], [43]. Curiously, however, variance in CSP RNA sequences has been reported in moths and other insect species such as aphids, beetles, flies and locusts [44]–[52]. In these studies, RNA variance has been described for CSPs in various tissues such as antennae and legs, but the origin of such RNA diversity could never be explained due to the use of a pool of tissues. One possible reason for such RNA variance could be the existence of specific typo-RNA editing mechanisms tuned to CSPs in all various individual tissues and organisms.

Here, we described the identification of tissue-specific RNA mismatches in Chemosensory Protein (CSP) genes. We compared cDNAs of BmorCSP RNAs (CSPs from silkworm moth *B. mori*) from the antennae, legs, head, pheromone gland and wings from silkworm moth with the same individual genomic DNAs by Sanger sequencing. In addition to the identification of A-to-G mismatches (as indicators of A-to-I RNA editing), we also observed significant numbers of other types of nucleotide substitution from BmorCSP RNAs. Interestingly, tissue-specific patterns of RDDs were reported with the pheromone gland having much higher prevalence of RDDs than other tissues. We thus attempted to determine whether such base mismatches are related to altered BmorCSP protein isoforms by Western Blots and peptide mass spectrometry.

## Results

### BmorCSP genes have different structures and expression profiles

**Figure 1:**
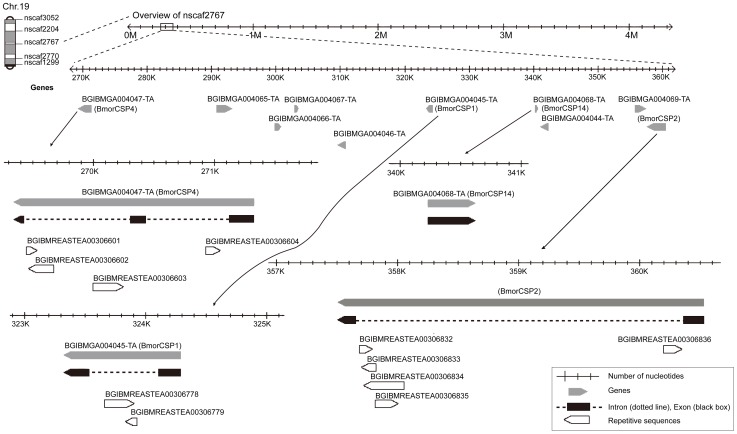
Schematic organization of BmorCSP genes. The four BmorCSPs (BmorCSP1, BmorCSP2, BmorCSP4 and BmorCSP14) matched scaffold nsaf2767 in positions 270K-360K. “BGIBMGA” are gene names identified for BmorCSPs in silkDB. Grey boxes indicate complete genes. The pointing arrow refers to the orientation of the gene (5′ to 3′). Exons are shown as black boxes and intron regions as dotted lines. Repetitive elements are shown as black-bordered white boxes. The numbers on the scale represent the position of the genes in the scaffold. *BmorCSP4* is the largest gene (1978 bps) and sits distantly from *BmorCSP1*, *BmorCSP2* and *BmorCSP14*.

Examination of the genome from the silkworm moth *B. mori* using bioinformatic tools shows that *BmorCSP1*, *BmorCSP2*, *BmorCSP4* and *BmorCSP14* genes co-exist on Chromosome 19 from the silkworm, separated only by 37670, 16033 and 16885 bps, respectively (Fig. 1). *BmorCSP1* and *BmorCSP2* are both single-introns. The two other genes selected for this study are either intronless (*BmorCSP14*) or double introns (*BmorCSP4*). The intron size and the number of retroposons among these genes differ greatly (Figure 1 & Table 1).

**Table 1:** Total number of RDDs on cDNA of *B. mori* CSP-RNAs depending on gene structure and tissue expression.

Gene	Size (bps)	*n* Introns (*n* retroposons)	Antennae	Legs	Head	Wings	Pheromone Gland	Total
*BmorCSP1*	964	1 (1)	14	6	9	5	27	61
*BmorCSP2*	3066	1 (0)	6	5	5	8	19	43
*BmorCSP4*	1978	2 (2)	12	14	1	2	9	38
*BmorCSP14*	363	0 (0)	20	13	16	17	25	91
			*52*	*38*	*31*	*32*	*80*	*233*

**Figure 2:**
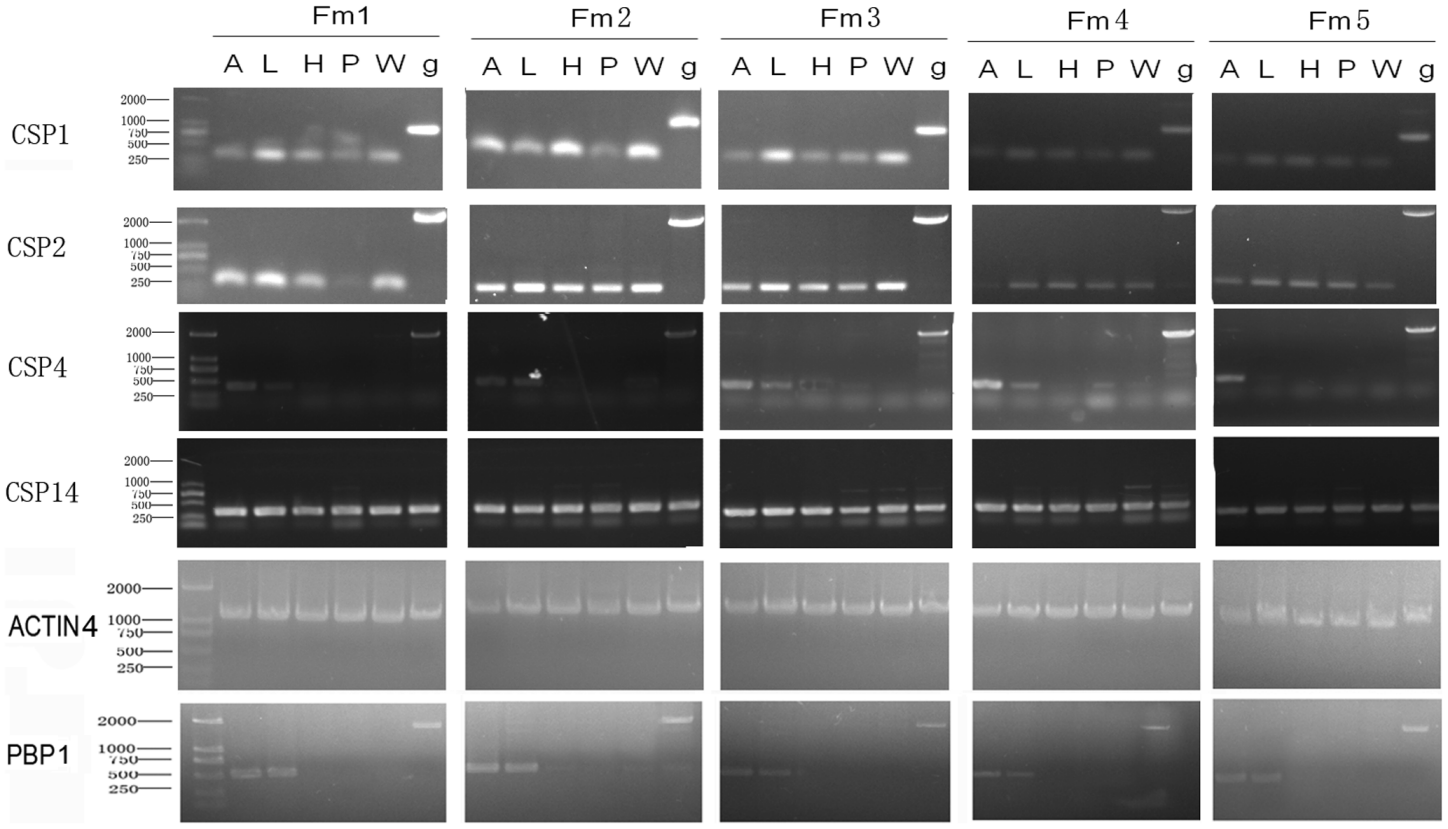
Individual tissue-expression of BmorCSP mRNAs. Agarose gel electrophoresis of BmorCSP1-, BmorCSP2-, BmorCSP4-, BmorCSP14-, PBP1- and Actin4-encoding cDNA PCR products from the antennae (A), legs (L), head (H), pheromone gland (P) and wings (W) from five individual newly-emerged virgin females of the silkworm moth *B. mori* (Fm1, Fm2, Fm3, Fm4 and Fm5). Genomic DNA (g) was amplified in the same conditions, showing no differences among individuals.

Using genomic DNA as template and BmorCSP1, BmorCSP2, BmorCSP4 or BmorCSP14 primers generated one single gDNA product that corresponded to the expected size for BmorCSP1, BmorCSP2, BmorCSP4 and BmorCSP14 genes, respectively. In addition to differences in gene structures, BmorCSP1, BmorCSP2, BmorCSP4 and BmorCSP14 genes showed differences in expression profiles. Although at different levels, all four genes expressed not only in the antennae but also in other parts of the silkworm body including legs, head, pheromone gland and wings (Figure 2).

The PCR reactions using cDNA as template also amplified PCR products of unique size (about 400–600 bps) in all tissue samples from each of the five individual females (Figure 2). BmorCSP1 was expressed in all tissues particularly in legs and wings. BmorCSP2 was expressed in the antennae, legs, head, pheromone gland and wings with no apparent differences. BmorCSP4 was preferentially expressed in the antennae and legs. Only minor amounts of BmorCSP4 were detected in the other tissues. BmorCSP14 was equally expressed in all tissues similarly to control Actin gene. PBP1 gene expressed solely in antennae and legs (Figure 2).

### A variety type of “RNA editing” is found in BmorCSP genes

We cloned and sequenced CSP-encoding cDNAs from the antennae, legs, head, pheromone gland and wings derived from each individual female, obtained genomic DNA sequences from the same individuals and compared the two.

**Figure 3:**
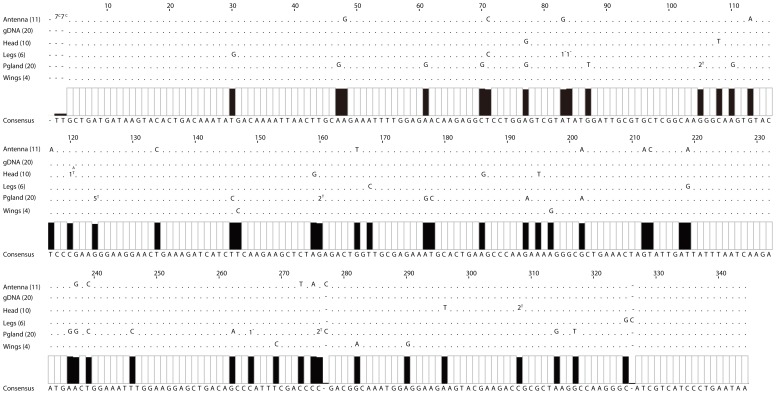
Tissue-specific editing on cDNA of BmorCSP mRNAs. Sequence analysis of cDNA PCR products encoding BmorCSP1 reveals nucleotide insertion, deletion and substitution at the editing sites (black rectangles) in the antennae, head, legs, pheromone gland (Pgland) and wings. The size of the black rectangle is proportional to the frequency of RDDs at this location. The consensus sequence below the alignment corresponds to the nucleotide composition of the genomic DNA sequence (gDNA) encoding BmorCSP1. The number in brackets next to tissue indicates the number of CSP clones obtained for each tissue cDNA and gDNA. “.” indicates that the base is similar to the consensus sequence on this location. “A”, “T”, “G”, “C” point out a switch to adenosine, thymidine, guanosine and cytosine base in tissue-specific cDNA sequences, respectively. “1^−^” indicates base deletion in one sequence of the tissue group. “n^A^” indicates switch to A in n sequences of the tissue group. Number of mismatches seen in only one tissue: 38. Number of mismatches seen in two tissues: 4.

Differences between cDNA and genomic DNA revealed an extremely high number of RDDs and/or specific typo RNA editing sites in all four CSP genes (Figure 3, Figure S1 & Table S1).

Cloning and sequencing of cDNA products identified PCR products as BmorCSP1, BmorCSP2, BmorCSP4 and BmorCSP14, respectively (NM_001043587, NM_001043715, NM_001098310 and NM_001043599). The cDNA sequences included initial codon (BmorCSP4 and BmorCSP14) or codon or the first amino acid of the mature protein (BmorCSP1 and BmorCSP2) and stop codon. They corresponded therefore to the entire BmorCSP1, BmorCSP2, BmorCSP4 and BmorCSP14 protein sequence produced by five different tissues (antennae, legs, head, pheromone gland and wings) in each of the five individuals (Fm1-5). We used the coding region from gDNA clones to compare gDNA and cDNA. Sequencing ten to twenty gDNA clones per individual shows no variation, but comparing nucleotide sequences from cDNA and genomic DNA encoding BmorCSP1, BmorCSP2, BmorCSP4 and BmorCSP14 identified a significantly high number of RDDs (Figure 3, Figures S1, S2 & Table S1). These RDDs were not necessarily due to errors during the *in vitro* reverse transcription reaction. Eleven cDNA samples (F-A2, F-L2, F-H2, F-W2, F-W4, F-A1, F-L1, F-W1, F-H2, F-L3 and F-A5) showed no RDD. All other cDNA samples showed RDDs, particularly the pheromone gland cDNA samples (Table S1).

We checked for the number of RDDs for other genes such as Actin and PBP1. We found a high level of RDDs in antennal and leg samples for the PBP1 gene, but comparatively, low rate of RDDs for the Actin gene (Figures S3, S4 & Table S2). Sequencing 499 bps-long PBP1 clones from antennal and leg samples showed a rate of 4 to 8.4, respectively, and 50% of A>G RDDs. Sequencing 1.130 Kb-long Actin cDNA sequences from all individual tissue samples identified a rate of RDDs of only about 0.09–0.8. After sequencing ten clones per tissue, the number of A>G RDDs on Actin was limited to 46 (T>C: 27, G>A: 14, C>T: 20 and G>T: 3).

The analysis of genomic and cDNA sequences encoding BmorCSP1, BmorCSP2, BmorCSP4 and BmorCSP14 in various tissues of *B. mori* female individuals identified five types of RDDs: 1. RDDs that produced subtle amino acid changes, 2. RDDs that changed an amino acid to a stop codon and produce shortened protein, 3. RDDs that induced early-stop codon position, 4. RDDs that induced late-stop codon and 5. RDDs that produced protein lacking specific amino acid motifs (Figure 3, Figures S1, S2 & Table S1). No changes of an amino acid to a stop codon and induction of shortened protein (lack of C-terminus) and no multiple codons deletion were detected on Actin (Figure S3 & Table S2). These types of RDDs were observed on PBP1 (Figure S4 & Table S2). Also interestingly, most RDDs on CSPs preferentially targeted bases neighbored by a 3′-adenosine (Figure S1). In addition, many without effects (silent) RDDs were found on BmorCSP1, BmorCSP4 and BmorCSP14 irrespective of tissues and variously on both apolar and polar residues as well as on the chiral amino acid glycine. In contrast, silent RDDs on BmorCSP2 were only a few and mainly located on polar site residues (Table S1).

Edited and unedited transcripts co-existed in nearly all individual tissue samples. Among these edited transcripts were sequences leading to new proteins via premature stop codon or amino acid change in specific tissues (Table 2). One C deletion at base 307 in the codon that translated His-87 of BmorCSP1 from the pheromone gland leads to an exchange of His to Ile and moves stop codon to base 433 resulting in a lengthener protein of 14.35 kDa with 18 extra-amino acids (Figure 3, Table 2, Figure S1 & Table S1). One deletion of A at base 187 results in a change of the stop codon position yielding a BmorCSP2 protein with prominent extra-tail in the antennae. Other BmorCSP2-RDDs observed in wings result in removal of specific motifs such as 45-DKIP-48 in the middle of the protein (Table 2, Figures S1, S2A & Table S1). One deletion of T base at position 215 leads to an exchange of motifs in the residues 51–72 (LQSDCNKCSDKQRENADAWIEF to YKATAINVATSNGSTPTLGLNL) in protein BmorCSP4 lacking of the entire C-terminal tail domain. This RDD represented 70% of the population of BmorCSP4 transcripts. It was identified in legs, head, pheromone gland and wings but not in the antennae (Table 2, Figures S1, S2B & Table S1). One G deletion at base 213 produces a truncated BmorCSP14 protein (8.3 kDa) lacking the last 36 amino acid residues in legs. A multi-bases deletion in wings leads to protein BmorCSP14 without the 37–45 amino acid motif YVDCLLGKG (Table 2, Figures S1, S2C & Table S1).

**Table 2:** Protein variations induced by specific RDDs and frame-shifts on cDNA of CSP RNAs.

Clone (tissue, individual)	RDD (base)	RNA Variant	Protein Variant	Amino Acids	Molecular Weight (kDa)
***BmorCSP1***				111	12.90
CSP1-PG1-3	- C 307	r.307c<	fsHis87*l	129	14.35
CSP1-A4-4	+ A 317	r.317a>	fsPro90*e	102	12.09
CSP1-PG5-5	+ T 318	r.318t>	fsAsp91*e	102	12.04
CSP1-Hd4-6	A/T 337	r.337a>t	pmutLys96*	96	11.00
CSP1-L5-8	-TA 115 116	r.115_116at<	fsMet27*e	58	7.00
CSP1-PG1-4	G/T 202	r.202g>t	pmutLeu51*	51	6.10
CSP1-L4-8	T/G 72	r.72t>g	pmutTyr23*	23†	2.90†
***BmorCSP2***				104	12.05
CSP2-A2-5	- A 187	r.187a<	fsIle47*l	104	12.37
CSP2-W2-8	C/T 331	r.331c>t	pmutLys93*	94	10.81
CSP2-W4-4	-G---A 181-192	r.181-192ga<	deleAsp45-Pro48	100	11.61
***BmorCSP4***				114	13.23
CSP4-L2-9	G/A 306	r.306g>a	pmutTrp81*	81	9.40
CSP4-PG4-6	- T 215	r.215t<	fsLeu51*e	74	8.26
CSP4-Hd4-14	- T 215	r.215t<	fsLeu51*e	74	8.26
CSP4-L5-11	- T 215	r.215t<	fsLeu51*e	73	8.11
CSP4-L3-14	- T 215	r.215t<	fsLeu51*e	73	8.11
CSP4-W4-4	- T215	r.215t<	fsLeu51*e	73	8.11
CSP4-A4-8	-A 164	r.164a<	fsGlu34*e	43	5.03
CSP4-A4-6	C/A 68	r.68c>a	pmutThr1*	22†	2.51†
***BmorCSP14***				109	12.35
CSP14-PG3-22	G/A 275	r.275g>a	fsAla85*e	89	9.00
CSP14-L3-7	- G 213	r.213g<	fsLys61*e	73	8.30
CSP14-Hd1-5	-CT 184 186	r.184_186ct<	pmutAla50*	50	5.60
CSP14-W4-24	-C---T 109-135	r.109-135ct<	deleTyr37-Gly45	100	11.40

RNA and protein variants are named following the nomenclature recommendations from Den Dunnen and Antonarakis [53]. “r” indicates that the base change occurs at the RNA level. “r.68c>a” denotes that at nucleotide 68 a C is mutated to an A. “r.115_116at<” denotes that at nucleotide 115 and 116 an A and a T are deleted. “r.181-192ga<” denotes that between nucleotide 181 (a G) and 192 (an A) all nucleotides are deleted. “p” is for protein. “pmutLys96*” denotes that amino acid Lysine-96 is changed to a stop codon (*). “fsPro90*e” denotes a frame shifting change with Proline-90 as the first affected amino acid and creation of an early stop codon (*e). “fsHis87*l” denotes a frame shifting change with Histidine-87 as the first affected amino acid and creation of a late stop codon (*l). “deleAsp45-Pro48” means that amino acids Asparagine-45 to Proline-48 are deleted following mutation on Asparagine 45. Pmut: Point mutation, fs: frame-shift,

† : Signal peptide. RDDs such as r.115_116at<, r.202g>t, r.72t>g, r.215t<, r.164a, r.68c>a, r.213g< and r.184_186ct< are probably short aborted transcripts processed by the cell and deleted.

### BmorCSP-RDDs are mainly found in the pheromone gland

In total, 91 RDDs were identified on cDNA of BmorCSP14 RNA, which was about twice more than the number of RDDs observed on cDNA of other BmorCSP RNAs (Table 1). Using hclust in R as a tool for phylogenetic analysis and tissue-distribution, the height of RDDs on BmorCSP14 was found to be highest in the wings, while the height of RDDs on BmorCSP4 was found to be rather high in legs (Figure S5). However, a significantly high level of RDDs was detected on BmorCSP1 and BmorCSP2 in the pheromone gland (about two to three times more than in other tissues; Figure S5). The female F1 where not a single RDD was detected on BmorCSP2 in the antennae, legs and wings has now displayed eleven RDDs on BmorCSP2 in the pheromone gland (Figure S1 & Table S1). Totally, more than eighty CSP-RDDs were detected in the pheromone gland tissue of the female silkworm moth (Table 1). The pheromone gland was *truffée* of RDDs in the four CSP genes investigated but not in Actin (Figures S1, S2, S3 & Tables S1, S2). RDDs in PBP1 were mainly found in legs (Figure S4 & Table S2). The height of RDDs in Actin was mostly tuned to antennal samples. It was in a range of 30–44 across all tissues. The height of RDDs in CSPs across all tissues was in a range of 5–25, 0–25 and 5–30 (Figure S5).

### BmorCSP-RDDs lead to changed proteins

A few RDDs identified on cDNA of RNA coding for BmorCSP1, BmorCSP2, BmorCSP4 and BmorCSP14 gave shortened proteins (of about 2–5 kDa) having only the signal peptide or a quarter of the protein because premature stop codon formed. Interestingly, most of the RDDs resulted in truncated proteins of about 6–10 kDa mainly in the legs, wings and pheromone gland (Table 2).

**Figure 4:**
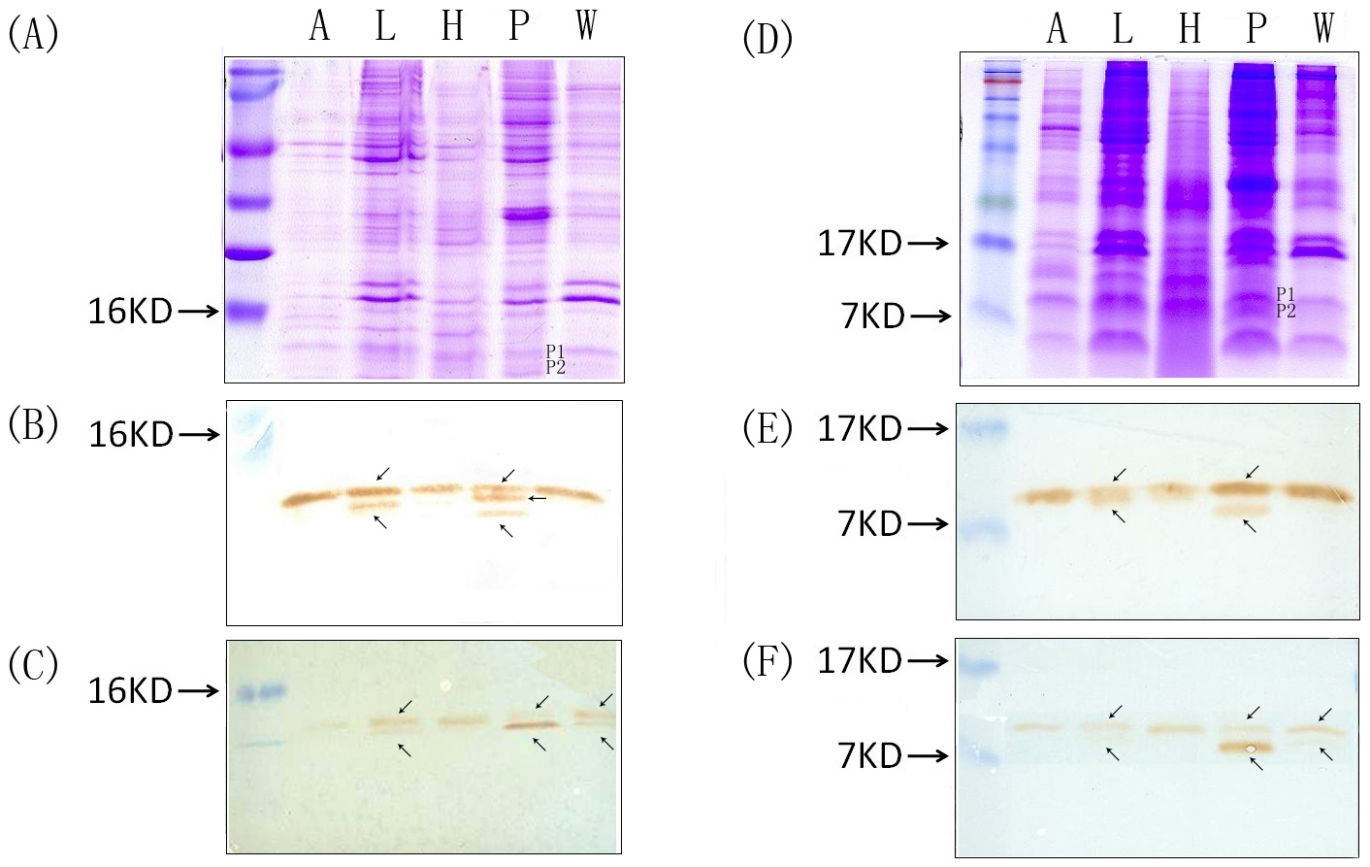
Functional expression of CSP-RDDs. Electrophoretic separation and Western blot analysis of CSP proteins in the antennae (A), legs (L), heads (H), pheromone gland (P) and wings (W) of female *B. mori*. Protein extracts corresponding to 10 antennae, 30 legs, 5 heads, 5 pheromone glands and 20 wings equivalent were subjected to 15% SDS-PAGE (A. and D.) and transferred to nitrocellulose membranes (B–C. and E.F.). Nitrocellulose blots were labeled with two different antisera: “anti-CSP1” (B. and E.) and “antiCSP14” (C. and F.). Positions of molecular weight markers are indicated on the left of the gel. Proteins of 9 to 14 kDa are labeled with the two CSP antibodies in the pheromone gland, legs and wings samples.

We thus checked for the expression of CSPs in different sizes in the female adult tissues of the silkworm moth *B. mori* (Figure 4). Using Western blot and two specific CSP-antibodies (made against “CSP1” and “CSP14” orthologs), we observed the presence of native CSPs together with shorter fragments (a size between 7 and 10 kDa) in the legs, wings and pheromone gland protein samples. Using both antibodies, only one protein band of about 12–13 kDa was detected in the antennal and head samples.

**Figure 5:**
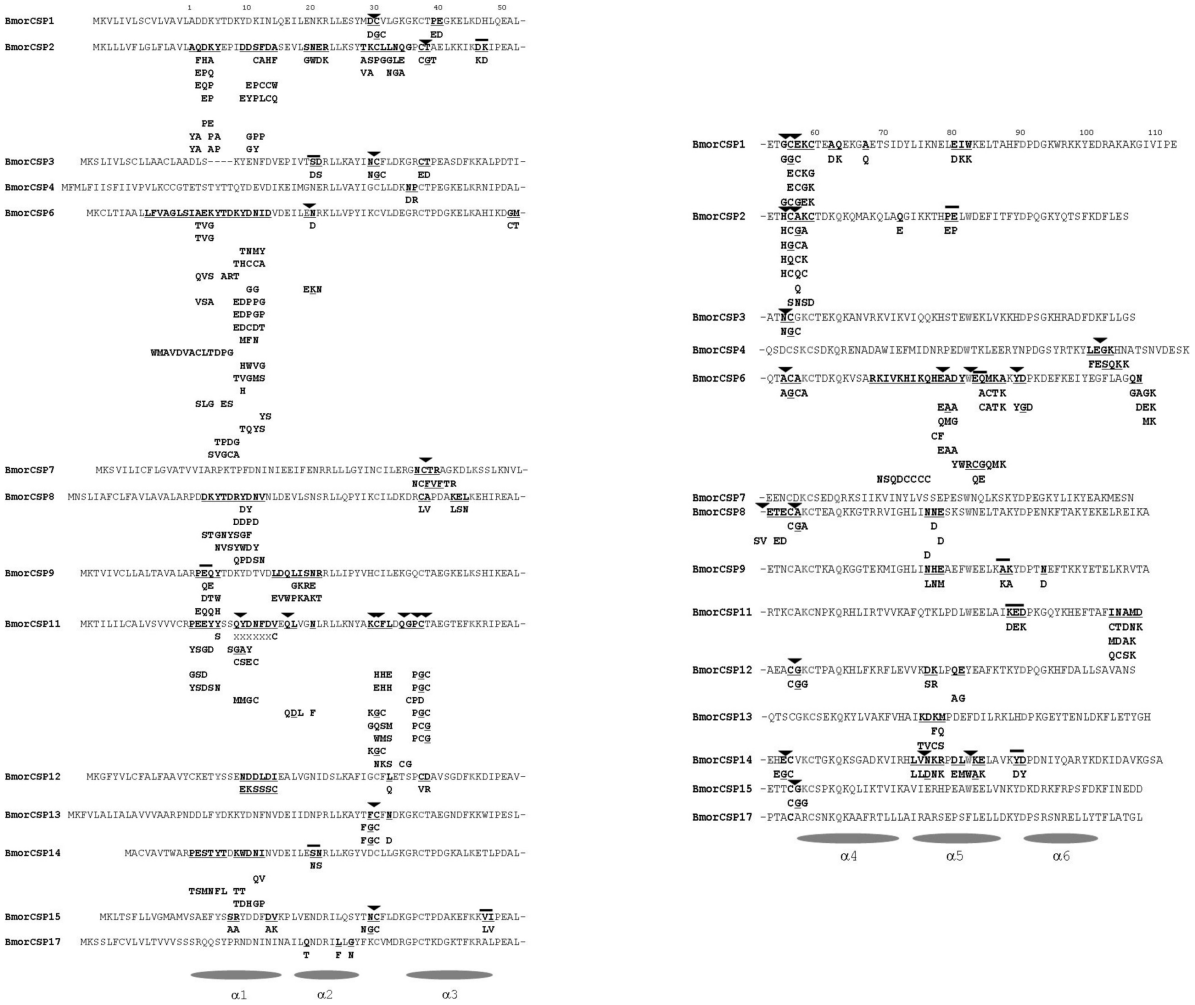
Amino acid mutations on BmorCSPs in the pheromone gland. Alignment shows the amino acid composition of proteins encoded by genomic DNA. Conserved amino acid residues are shown in grey. Mutations sites on the native protein are underlined. Mutant peptide motifs are shown in bold. The arrowhead indicates amino acid insertion (mainly G). The dash above residues indicates amino acid inversion. The cross (x) indicates amino acid deletion in the motif. The grey circles below the alignment indicate the position of functional elements such as α-helices [36].

The two immunoreactive bands corresponding to shorter CSP fragments (P1 and P2) were subjected to liquid chromatography coupled to tandem mass spectrometry analysis (Figures 4–5 & Tables S3, S4). A high number of peptides with amino acid insertion, inversion, truncation and/or replacement were discovered for BmorCSP1, BmorCSP2, BmorCSP3, BmorCSP4, BmorCSP6, BmorCSP7, BmorCSP8, BmorCSP9, BmorCSP11, BmorCSP12, BmorCSP13, BmorCSP14, BmorCSP15 and BmorCSP17 in the pheromone gland (Figure 5 & Tables S3, S4). Most notably, glycine residue insertion was observed before or after cysteines at position 30, 37 and 56. A particularly high number of mutations in the first fifteen residues of the N-terminus led to multiple protein variants for BmorCSP2, BmorCSP6 and BmorCSP11 (Figure 5).

## Discussion

We chose a PCR approach and Sanger sequencing of about ten clones per PCR product to analyze differences between gDNA and cDNA encoding four CSPs in five tissues from five single individual female *B. mori* silkworm moths. The proteins BmorCSP1, BmorCSP2, BmorCSP4 and BmorCSP14 show about 32–42% identity between each other. Taq polymerase shows mistake in a range of 1/10000 nucleotides in particularly in low abundant products and products exceeding 1 kb. Our study deals with high abundant products not exceeding 300 bps. The error rate reported for Taq is very much dependent on the assay used. Using the same reverse transcription/PCR assay, dNTP concentration (10 mM), Taq polymerase, PCR conditions, cloning and sequencing strategy, we found that genomic DNA showed no variation. However, some cDNA had no RDD (F-A2, F-L2, F-H2 and F-W4 for CSP1; F-A1, F-L1, F-W1, F-H2, F-L3 and F-A5 for CSP2; F-W2 and F-P4 for CSP14), but most of the samples had a load of RDDs depending on the individual and the tissue (see Table S1).

We show a particularly high number of RDDs for the gene BmorCSP4 that retains two retroposons. Insertion of retroposon may boost RDDs and mRNA variance (see Figure 1 & Table 1). Most importantly, we show also a number of RDDs for the three other genes BmorCSP1, BmorCSP2 and BmorCSP14 in many various tissues such as the antennae, legs, pheromone gland, head and wings. BmorCSP1, BmorCSP2 and BmorCSP14 are expressed throughout the whole insect body, while expression of BmorCSP4 is more restricted to the antennae and legs (see Figure 2). We found a correlation between the number of RDDs and tissue-specificity (see Table 1, Figures 3–4, Figures S1, S2, S3, S4, S5 & Table S1), thus suggesting that the number of RDDs is correlated to the function(s) the CSP protein has in each tissue.

The extremely high number of CSP-RDDs in the pheromone gland suggests an important function of CSPs in pheromone biosynthesis (see Table 1). CSPs are known to interact with long chain fatty acyl pheromone precursors [34]. Noctuid species such as *Heliothis virescens* that use multicomponent pheromones are known to express multiple CSPs in the pheromone gland [32]. In contrast to *Heliothis*, the pheromone from the silkworm moth *B. mori* is only made of two chemicals, Bombykol and Bombykal [54], [55]. Therefore, the accumulation of CSP variants in both *Heliothis* and *Bombyx* suggests that CSPs bind to compounds in the first steps of pheromone biosynthesis. The two bombycid pheromone compounds are constructed from triglycerides (hexadecenoic, hexadecadienoic, oleic, linoleic and linolenic acids) accumulated in lipid droplets within the cytoplasm of the pheromone gland cells [56]. Thus, CSPs could help serve the transport and accumulation of triglycerides in the gland until the onset of pheromone production. Inter-individual variations in pheromone production have been described in bark beetles and moths [57]. It would be interesting to check in various insect species whether a pheromone gland without RDDs on CSP genes produces the same amounts of pheromone than a pheromone gland *truffée* of RDDs in this gene family.

We do not exclude that some of these hundreds RDDs represent allelic variations, transcriptase mistakes or polymerase errors. However, it is important to note that we found a high number of A>G mismatches (116) compared to T>C (48), G>A (41), C>T (30) and G>T (19) as found for RNA editing on specific elements from the human transcriptome [58], [59]. We found that 66 RDDs are synonymous, but more than 200 RDDs are non-synonymous. In analyzing human RNA editing, most studies suggested that there are very few non-A-to-G RDDs and other types of RDDs are largely artifacts [60]–[62]. In humans, the majority of RNA editing occurs in non-coding regions of the transcriptome [63]. Analyzing moth RNA editing for a specific gene family such as CSPs, we show that there are a lot of non-A-to-G RDDs in coding regions of the transcriptome of various individual tissues, particularly the pheromone gland. We found tissue-specificity in variety types of RDDs on cDNAs of RNA encoding BmorCSPs and truncated BmorCSP products both mainly in the pheromone gland (see Figures 3, 4, 5 & Figures S1, S2, S3, S4, S5). A-to-G mismatch catalyzed by members of adenosine deaminase (ADAR) enzyme family is structure-dependent [64]. Using CentroidFold (www.ncrna.org) identifies a dozen of possible RNA duplex structures for our predicted A-to-G mismatches (not shown). The elements that led to specific cellular recognition of duplex RNA structures in the silkworm moth remain mysterious. However, multiple ADAR mRNA variants have been reported mainly in the embryos of *B. mori* (EF540770-AB183871). This may suggest the existence of tissue-specific ADARs similarly to our A-to-G mismatches.

Also interestingly, there are various pioneer biochemical studies that suggest the existence of multiple CSP protein variants originating from one single particular gene. Three BmorCSP1 proteins of 12.027–12.891 kDa sharing the same N-terminal sequence have been purified from the *Bombyx* antennae [17]. Similarly, two p10 protein isoforms have been extracted from cockroach tissues [21]. We have thus checked whether mutant proteins could result from RDDs in the RNA by Western blot and de novo peptide sequencing of immunoreactive protein bands (see Figures 4–5). Using two specific CSP-antibodies shows multiple forms of CSP with molecular weight in a range of 9–14 kDa in the pheromone gland, legs and wings in agreement with the hierarchical clustering of tissues based on RDDs (see Figure S5). Sequencing gDNA clones did not identify possible pseudo-genes for BmorCSP1, BmorCSP2, BmorCSP4 and BmorCSP14 as found by the genome sequencing approach on both Chinese and Japanese silkworms. CSP pseudo-genes are unexpressed [65]–[67]. Our Western blot result is also in better correlation with the size of proteins produced by RDDs on cDNA of CSP-RNAs than with the size of the various existing BmorCSPs (see Table 2). The *Bombyx mori* genome contains 20 genes that encode for some “short” (about 90 amino acids) and “long” (more than 200 amino acids) CSPs [40]. All of these BmorCSPs have a size (including the signal peptide) comprised between 13.27 and 24.65 kDa and a size (without the signal peptide) comprised between 10.96 and 14.69 kDa [17], [40]. In addition, we only see products of the predicted CSP size and smaller on the western blots and no larger products. Correlatively, we observe essentially non-sense mutations and frame-shifts that are expected to produce premature terminations but comparatively only a small number of frame-shifts expected to produce larger proteins. We did not observe RDDs leading to proteins that are larger than 14.35 kDa (see Table 2). These observations together with tissue-specificity of both RDDs and truncated protein isoforms strongly suggest that RDDs result in protein variants of different sizes and shapes in specific tissues of the silkworm moth *B. mori*.

We demonstrate the existence of multiple mutant BmorCSP proteins in the pheromone gland. Mutations on the peptide sequence are mainly found in the N-terminus, α2 and α3-helices, the loop region between α3 and α4-helices, α5-helix and the free C-terminus (see Figure 5). Mutation on the motif sequence KYLEGKH from BmorCSP4 led to a YFESQKK sequence very similar to *Tribolium castaneum* CSP4 (#NP_001039285; 86% identity). Similarly, many other mutant peptide fragments such as LVPDALSNK, ALSVEEDCAK, KLLVPYLK, WMAVDVACLTDPGYDNLDVDELLDQR, SLGYESKYDNLDVEELLENR, TQYSDVDELLENR, VLRHLLDNKPEMWAK and LLTNDRLFLNYFK from BmorCSP6, BmorCSP8, BmorCSP14 or BmorCSP17 were very similar to fragments of CSPs identified in some beetle, bug, butterfly, fly and locust species (#AAC25400, AGD80085, AGY49267, AGY49271, BAF91717, CBA11329, EHJ70185, NP_524966, NP_001039286 and NP_001039288; >70% identity). The peptide fragment TQLSRPEDVK from the P2 library showed no significant analogy with BmorCSPs but was significantly related to *Aphis gossypii* CSP7 (#AGE97646; 70% identity). AgosCSP7 is related to BmorCSP19 (57% identity). Mutant CSP peptides probably exist in all various insect species. In all, about a hundred mutant CSP fragments have been identified in the P1 and P2 de novo peptide library of the pheromone gland from the female silkworm (see Tables S3, S4).

RNA editing is therefore particularly important to expand drastically the repertoire of the CSP gene family. If RDDs have potential to multiply for instance hundred times the number of CSPs, not 20 but 2000 CSP proteins could be produced through RNA editing in the silkworm. A model for CSP synthesis based on RDDs is proposed on figure S6. In this model, RNA editing yields a very high number of proteins from a very limited number of genes. We did not find high substitution rates on RNA coding for Actin, but we found high substitution rates on pheromone-binding protein (PBP) RNA (see Table S2). In addition, we found a high number of mutant peptides corresponding to odorant-binding proteins such as OBP6, general OBP2, general OBP56d, PBP-related protein 3, sericotropin and protein B1 (see Tables S3, S4). RNA editing could be an important mechanism for all sensory gene families.

If the additional load of CSP/PBP/OBP RNA transcripts within the cells has no function, then it surely can provide the raw material for evolution to work with. The eukaryotic cells must deal with a lot of junk DNA (introns). We think that the eukaryotic cells must also deal with a lot of junk RNA. Our study in the *Bombyx* suggests that RNA editing is not restricted to Human but probably exist in all various organisms for many gene families. With such a high mutation rate on the RNA, the cell gains a high degree of probability to produce quickly a beneficial mutation, much more than in a situation where the mutation rate is lower or where mutator mechanisms are triggered only in response to certain selective forces. We even speculate that RDDs precede selective evolutionary pressures and all possible changes in the environment. Under these circumstances, the moth gains a strong advantage in case the environment changes. RNA is already ready to produce proteins with crucial amino acid changes in response to crucial changes in the environment.

RNA-seq and proteome analysis of tissue samples from the silkworm moth *B. mori* are now required to clarify which mutant proteins result from RDDs in the RNA. Messenger RNA transcripts have a different fate and probably not all mRNAs will be transcribed into proteins [68]. In mammals and yeasts, diverse RNA-binding proteins interact with functionally related sets of RNA to decide the excision or translation of mRNAs and the number of protein variants in one single tissue [68], [69]. Our study shows that similar mechanisms exist in the moth pheromone system and that they are tightly regulated in a cell tissue-specific manner. We propose to open a new field of research that is to investigate the key regulatory mechanisms of RNA editing underlying pheromone production and reception in insects. This may be important not only to solve the complex machinery of protein expression within eukaryotic cells but also to understand genetic regulation in a highly specific chemosensory system.

## Methods

### Ethics statement

“N/A”

### Silkworm rearing

Silkworm *B. mori* cocoons (Qingsong x Haoyue crossbreeds) come from institute colonies (Yantai) reared from eggs in insect growth chambers maintained at 23°C with 70% relative humidity and L15:D9 photoperiod. Nymphs were sexed and maintained at room temperature in ambient environment until adult eclosion. Five females emerging from the same batch were immediately isolated, kept unmated and dissected in mid-photophase for tissue extraction (antennae, legs, pheromone gland, wings and head). Dissected tissues were immediately frozen and stored at −80°C until genomic DNA and RNA were extracted.

### Analysis of silkworm genome

The genomic DNA sequence of four *B. mori* CSP genes was extracted from the available silkworm genome at http://www.sgp.dna.affrc.go.jp/KAIKObase/ [65]. Genomic sequences coding for BmorCSP1, BmorCSP2, BmorCSP4 and BmorCSP14 were retrieved by blasting the nucleotide sequence of mRNA (BmorCSP1, #AF509239, AJ93410, AY766376, DQ855510, FJ425876, NM_001043587; BmorCSP2, #AF509238, DQ855519, AJ973407, NM_001043715; BmorCSP4, #NM_001098310; BmorCSP14, #AB243746, DQ855516, NM_001043599) against the assembly of scaffolds and contigs stored in the KAIKobase. Genomic sequences encoding BmorCSP1, BmorCSP2, BmorCSP4 and BmorCSP14 were all found in the scaffold 36 (contigs BABH01021425, BABH01021427, BABH01021421 and BABH01021426, respectively). Similar analysis was performed using the SilkBase at http://www.silkdb.org/silkdb/ [66], [67]. In the SilkBase, BmorCSP1, BmorCSP4 and BmorCSP14 genomic sequences were found in the scaffold nscaf2767 (contigs BGIBMGA004068-TA, BGIBMGA004047-TA and BGIBMGA004068, respectively). BmorCSP2 was found to be unannotated. The BmorCSP2 gene was retrieved using the tBLASTn algorithm in the protein db silkworm annotated protein and the nucleotide db silkworm genome assembly from the SilkBase. Two exons were found spanning over 357504–357683 and 360388–360519 bases, respectively (see Figure 1). Coding (exon) and non-coding (intron) sequences were identified by plotting the CSP-cDNA nucleotide sequence against the scaffold or contig open reading frame (searchlauncher algorithm). To check the presence of retroposons in the intron, the intron sequence was simply subjected to the BLASTn or tBLASTx algorithm (NCBI).

### Molecular characterization of individual genomic DNA and mRNA

For each individual female, genomic DNA was extracted from the abdomen and thorax following the Easy DNA extraction protocol (Qiagen). Total RNA was extracted from the rest of the body (e.g. antennae, legs, pheromone gland, wings and head) using the TrizolTM method (Life Technologies). First strand cDNAs of each tissue RNA were synthesized using the Boehringer kit for high-fidelity retrotranscription following the supplier's recommendation. Antennal, leg, pheromone gland, head and wing cDNA as well as genomic DNA aliquots (1 ng/µl) from each of the five female individuals were then used as templates in PCRs employing High-Fidelity (HIFI) Taq polymerase (Transgen), dNTPs (10 mM) and oligonucleotide primers designed against the 5′ and 3′ ends of Actin4, PBP1 and four specific CSP genes (BmorActin4, #BMU49644; BmorPBP1, #X94987, NP_001037494.1; BmorCSP1, #AF509239, AJ93410, AY766376, DQ855510, FJ425876, NM_001043587; BmorCSP2, #AF509238, DQ855519, AJ973407, NM_001043715; BmorCSP4, #NM_00109831; BmorCSP14, #AB243746, DQ855516, NM_001043599). Nucleotide sequences of primers were as follows:

BmorCSP1s 5′-TTGCTGATGATAAGTACACTGACA-3′,

BmorCSP1as 5′-TTATTCAGGGATGACGATGC-3′,

BmorCSP2s 5′-CAAGACAAATACGAACCAATC-3′,

BmorCSP2as 5′-TTAAGACTCAAGAAAATCTTTG-3′,

BmorCSP4s 5′-ATGTTTATGCTCTTCATAATTTC-3′,

BmorCSP4as 5′-TTATTTAGATTCATCAACGTTGC-3′,

BmorCSP14s 5′-ATGGCATGTGTGGCGGTC-3′,

BmorCSP14as 5′-CTAAGCGGAGCCTTTGACG-3′

BmorPBP1s: 5′-ATGTCTATCCAAGGACAGAT-3′

BmorPBP1as: 5′-ATTCTCAAACTTCAGCTAAAAT-3′

BmorActin4s: 5′-ATGTGCGACGAAGAAGTTGCCGCGTT-3′

BmorActin4as: 5′-TTAGAAGCACTTCCTGTGTA-3′

Primers tuned to signal peptide of BmorCSP1 did not amplify any PCR products (data not shown). PCR cyclers were programmed with annealing temperature depending on the Tm of primers: 94°C for 5 min, then 35 cycles of 94°C for 30 sec, 50–58°C for 30 sec and 72°C for 1 min followed by and extension step of 72°C for 5 min. For control experiments on BmorCSP14, two reverse transcription reactions were performed, one lacking the reverse transcriptase. PCR was done on both samples using specific CSP14 primers. There was no band in the reaction for the reverse transcription with no enzyme, attesting to the absence of genomic DNA contamination in RNA samples (Figure S7). Both cDNA and gDNA PCR products were purified using GENECLEAN® kit (Qbiogene, Inc.) and cloned into pEASY-T1 (Transgen) or pGEM-T Easy vector (Promega) following the manufacturer's recommendations. Sequencing of cDNA and gDNA clones was performed using the Big Dye terminator v3.1 cycle sequencing kit (Applied Biosystems) and vector specific primers. Sequencing products were purified by isopropanol precipitation and subjected to the ABI3100 DNA sequencer (Applied Biosystems sequencing platform). Using this approach, cDNA and gDNA clones encoding BmorActin4, BmorPBP1, BmorCSP1, BmorCSP2, BmorCSP4 and BmorCSP14 in five different tissues in five female individuals were obtained. Sequence information was assembled using Sequencher 3.0 software (Gene Codes Corporation) and used for comparative analysis (Mega 4.0). All sequencing data has been deposited in GenBank (see Tables S1, S2).

### Computational analysis of cDNA and gDNA sequences

The cDNA and gDNA sequences from different tissues for each gene were aligned separately. All the DNA sequences for each of the four CSPs and Actin genes including duplicate copies were aligned using MUSCLE [70]. Correspondence of cDNA positions to those of gDNA sequences identified specific RDDs. A consensus sequence for each gene alignment for all tissues was generated using Jalview [71]. The aligned sequences for each gene were further subdivided into separate tissue alignment. This tissue alignment was then compared to the consensus sequence. The base difference in each position of the alignment was classified as no change, insertion, deletion and substitution position. Total number of changes was counted for all the alignment positions. A matrix summarizing different substitutions for each tissue was prepared from the above counts. This matrix was then used to generate a hierarchical clustering tree for each gene. This was repeated for all genes and for all tissues.

Hierarchical clustering analysis of tissue-specific cDNA and gDNA sequences was performed using the hclust command in R an open source statistical package. Sequences without RDDs were excluded from the analysis. The summarized matrix now contained only the RDD sites between the tissues. This matrix built by clustering RDDs (substitution, deletion, and insertion) was used to compute pairwise distances (euclidean distances in R) between the tissues for each CSP gene and Actin. The final phylogenetic tree was estimated with hclust command and default complete linkage method.

### Protein analysis

Total protein extracts were prepared from antennae, legs, head, pheromone gland and wings samples of five newly-emerged Qingsong x Haoyue *B. mori* females by grinding frozen tissues in a standard protein extraction buffer (20 mM Tris-HCl, PH 7.4). Protein homogenates were centrifuged at 10,000 g for 15 min. Cleared supernatants were transferred to clean tubes and stored at −80°C until electrophoresis and western blot experiments.

In electrophoresis experiments, extract sample loads of 15 µl were separated on 15% SDS-polyacrylamide gels ran at room temperature (JUNYI DONGFANG Electrophoresis Co. Ltd, China). In denaturing SDS-PAGE separation, proteins migrated according to size and no other parameter. For protein identification by Western blots, proteins were transferred to nitrocellulose membranes (Semi-Dry Transfer System, JUNYI DONGFANG, China) and subjected to immunological assay. In this assay, the membranes were blocked for 2 hrs in TBST buffer (20 mM Tris-HCl, pH 7.5, 150 mM NaCl, and 0.05% Tween 20) with 5% no-fat dried milk at room temperature. The membranes were then incubated with a 1∶1,000 dilution of primary antibody (anti- HarmCSP1b, orthologous to BmorCSP1, or anti- HarmCSP29, orthologous to BmorCSP14) for 2 hrs. After three washes with TBST buffer, the membranes were incubated with horseradish peroxidase (HRP)-conjugated goat anti-mouse antibody (1∶2,500) for another 2 hrs; visualization of immuno-labeled proteins was done using enhanced CAB stain kit (Tiangen, China).

### In gel digestion of SDS-PAGE separated proteins

Immunoreactive bands (P1 and P2) were cut from the gel and sliced into ca 1 mm^3^ pieces. Gel samples were destained with vortexing in 1∶1 acetonitrile (ACN):100 mM ammonium bicarbonate (ABC), reduced with 10 mM dithiothreitol (DTT) at 56°C and alkylated with 50 mM iodoacetamide (IAA) for 20 min in the dark. The gel pieces were dehydrated with ACN and dried under a hood. Overnight in-gel digestion at 37°C was carried out with 50 µl of 10 ng/µl of proteomics grade trypsin (Sigma) or Lys-C (Wako) with a prior 2 hrs pre-incubation on ice. Peptides were extracted from the gel with 5 min sonication, followed by 30 min vortexing in 2 volumes of 1∶2 5% formic acid (FA):ACN. The organic phase was removed in a vacuum-centrifuge and the peptide mixture purified on a C18 StageTip [72].

### Nano-LC/MS/MS analysis

For liquid chromatography coupled to tandem mass spectrometry analysis, peptides were separated on a Ultimate 3000 RSLCnano system (Dionex) using a cartridge trap-column in backflush configuration and an analytical 50 cm Easy-spray column (Thermo Fisher Scientific). Peptides were eluted at 200 nl/min with a 8–40% B gradient (buffer B: 80% ACN+0.1% FA, buffer A: 0.1% FA) to a Q Exactive MS/MS operating with a top-10 strategy and a maximum cycle time of 1 sec. Briefly, one 350–1400 m/z MS scan at a resolution setting of R = 70000 was followed by fragmentation of 10 most intense ions >+1 charge state at R = 17500. Dynamic exclusion was set to 20 sec.

### De novo peptide interpretation of mass spectra and peptide analysis

Raw data were transformed to .mgf peaklists and de novo interpreted with PEAKS 6 Client software (Bioinformatic Solutions). Only peptides with charge state 2–5 and with scan quality score >0.65 were subjected to analysis. Precursor and fragment mass tolerances were set to 5 ppm and 0.02 Da, respectively. Results were filtered for minimum Average Local Confidence score of 60, providing four peptide libraries (P1 Lys-C, P2 Lys-C, P1-Trypsin and P2-Trypsin). Ultimately, the amino acid sequences of the 1364 P1 Lys-C, 617 P2 Lys-C, 8439 P1-Trypsin and 9653 P2-Trypsin de novo peptides were subjected to BLASTp algorithm (NCBI) to search for analogy with CSPs. Analogous peptides (>50% identity) were aligned together with the native protein sequence encoded by genomic DNA.

## Supporting Information

Figure S1 Sequence analysis of gDNA and cDNA PCR products encoding CSPs in various tissues of five individual *B. mori* females (F1–5). The nucleotide sequences from genomic DNA (gDNA) of a single individual female are shown atop cDNA sequences without RDD from the same single individual female. Specific tissue/individual RDDs are shown below. Only a few examples of RDDs on cDNAs of RNA encoding BmorCSP1 (A), BmorCSP2 (B), BmorCSP4 (C) and BmorCSP14 (D) from the antennae (A4-4, A1–7, A2–5, A3–7, A4–6, A4–8, A2–4 and A1–16), legs (L4–8, L5–8, L2–9, L3–14, L5–11 and L3–7), head (H4–6, H4–14, H1–5), pheromone gland (P1–4, P5-5, P1–3, P4–6, P3–22) and wings (W2–8, W4-4, and W4–24) are represented. Nucleotide insertion, deletion and substitution at the editing sites (RDDs) are underlined. PmutAA: RDD and typo amino acid change, pmutAA*: RDD and change amino acid to stop codon, fsAA*e: Frame-shift RDD and switch to early stop codon position, fsAA*l: Frame-shift RDD and switch to late stop codon position, deleAA: Codon deletion, Pmut: Point mutation (RDD), fs: Frame-shift, *: Stop codon, e: early, l: late. Analyzing twenty cDNA sequences for each individual tissue sample, point mutations (RDDs) are found for all the four BmorCSP genes investigated in this study. No fsAA*e RDDs are found for BmorCSP2. No fsAA*l RDDs are found for BmorCSP4 and BmorCSP14. No deleAA RDDs are found for BmorCSP1 and BmorCSP4.
(TIF)

Figure S2 Tissue-specific editing on cDNA of BmorCSP2, BmorCSP4 and BmorCSP14 mRNAs. Sequence analysis of cDNA PCR products encoding BmorCSP2 (A.), BmorCSP4 (B.) and BmorCSP14 (C.) reveals nucleotide insertion, deletion and substitution at the editing sites (black rectangles) in the antennae, head, legs, pheromone gland (Pgland) and wings. The size of the black rectangle is proportional to the frequency of RDDs at this location. The consensus sequence below the alignment corresponds to the nucleotide composition of the genomic DNA sequence (gDNA) encoding BmorCSP2 (A.), BmorCSP4 (B.) and BmorCSP14 (C.). The number in brackets next to tissue indicates the number of CSP clones obtained for each tissue cDNA and gDNA. “.” indicates that the base is similar to the consensus sequence on this location. “A”, “T”, “G”, “C” point out a switch to adenosine, thymidine, guanosine and cytosine base in tissue-specific cDNA sequences, respectively. “1^−^” indicates base deletion in one sequence of the tissue group. “n^A^” indicates switch to A in n sequences of the tissue group. Number of mismatches seen in only one tissue: 31–62. Number of mismatches seen in two tissues: 1–7.
(TIF)

Figure S3 Tissue-specific editing on cDNA of Actin4 mRNAs. Number of mismatches seen in only one tissue: 92. Number of mismatches seen in two tissues: 1.
(TIF)

Figure S4 Tissue-specific editing on cDNA of PBP-1 mRNAs. Number of mismatches seen in only one tissue: 39. Number of mismatches seen in two tissues: 3.
(TIF)

Figure S5 Hierarchical clustering of tissues based on RDDs in BmorCSP and Actin genes (hclust command in R). The distance (height) indicates the number of RDDs in a given tissue.
(TIF)

Figure S6 Mutation mechanism of CSPs. Genomic DNA is transcribed into premature mRNA (1) that is processed for intron splicing and excision of non-coding region (2). The mature mRNA is then subjected to typo editing (3). This results in a switch of one or few nucleotides from which all various combinations are possible. One pinpointed mutation (left) in the codon for Tyrosine (UAU) on mRNA can be silent (UAC also codes for Tyr) or can replace Tyr by Asp (GAU), Asn (AAU), His (CAU), Cys (UGU), Phe (UUU), Ser (UCU) and Tyr UAC (Tyr). The substitution of U to A can lead to stop codon and abort CSP. Single base insertions or deletions (right) can induce a shift in the reading frame (frame-shift mutations) resulting in a drastic change in the CSP. The protein can be aborted or have a prominent extra C-terminal tail. Deletions that remove a few juxtaposed bases in the internal part of the CSP-RNA can produce shorter proteins lacking of specific motifs. Consequently, a high number of protein variants are produced from one single CSP gene, rejecting the dogmatic concept ‘One gene-One protein’.
(TIF)

Figure S7 Control experiments on BmorCSP14. Agarose gel electrophoresis of BmorCSP14 encoding cDNA PCR products from the antennae (A), legs (L), head (H), pheromone gland (P) and wings (W) from five individual newly-emerged virgin females of the silkworm moth, *B. mori* (Fm1, Fm2, Fm3, Fm4 and Fm5). +: RT reaction including the reverse transcriptase, −: RT reaction lacking the reverse transcriptase. No product was amplified in the RT reaction lacking the reverse transcriptase, demonstrating that there is no genomic DNA contamination in RNA samples.
(TIF)

Table S1 RDDs on cDNA of CSP-RNAs. Pmut: Point mutation, Fs: Frame-shift. pmutAA*: Changes an amino acid to a stop codon and induces shortened protein (lack of the C-terminus). fsAA*e: Leads to amino acid change and modification of stop codon position (early-stop codon). fsAA*l: Leads to amino acid change and modification of stop codon position (late-stop codon). deleAA: Deletes multiple codons and induces shortened protein (lack of central amino acid motifs). A: Antennae, L: Legs, Hd: Head, PG: Pheromone gland, Wg: Wings. F1–5: Individual female 1–5. ^+^: RDDs in the same tissue in different individuals; ^++^: RDDs in various tissues from the same individual; ^+++^: RDDs in various tissues from different individuals. RDDs on functional elements are bolded and underlined. §: F-L4 T>G on site 72 corresponds to RDD on the signal peptide. Sequencing more CSP14 clones from the (+) reaction identified even more RDDs (point mutations in italic).
(DOC)

Table S2 RDDs on cDNA of Actin and PBP-RNAs. Pmut: Point mutation, Fs: Frame-shift. fsAA*e: Leads to amino acid change and modification of stop codon position (early-stop codon). fsAA*l: Leads to amino acid change and modification of stop codon position (late-stop codon). No changes of an amino acid to a stop codon and induction of shortened protein (pmutAA* RDD) and no deleAA RDDs (deletes multiple codons and induces shortened protein lacking of central amino acid motifs) have been detected on Actin. A: Antennae, L: Legs, Hd: Head, PG: Pheromone gland, Wg: Wings. F1–5: Individual female 1–5. ^+^: RDDs in the same tissue in different individuals; ^++^: RDDs in various tissues from the same individual; ^+++^: RDDs in various tissues from different individuals.
(DOC)

Table S3 P1 de novo peptide sequences. Amino acid replacements are shown in bold and underlined.
(DOC)

Table S4 P2 de novo peptide sequences. Amino acid replacements are shown in bold and underlined.
(DOC)
