# Supplementary figures and images for: Molecular Evidence of RNA Editing in Bombyx Chemosensory Protein Family
Source: PLoS One. 2014 Feb 13;9(2):e86932. doi: 10.1371/journal.pone.0086932 (PMC3923736; doi:10.1371/journal.pone.0086932)

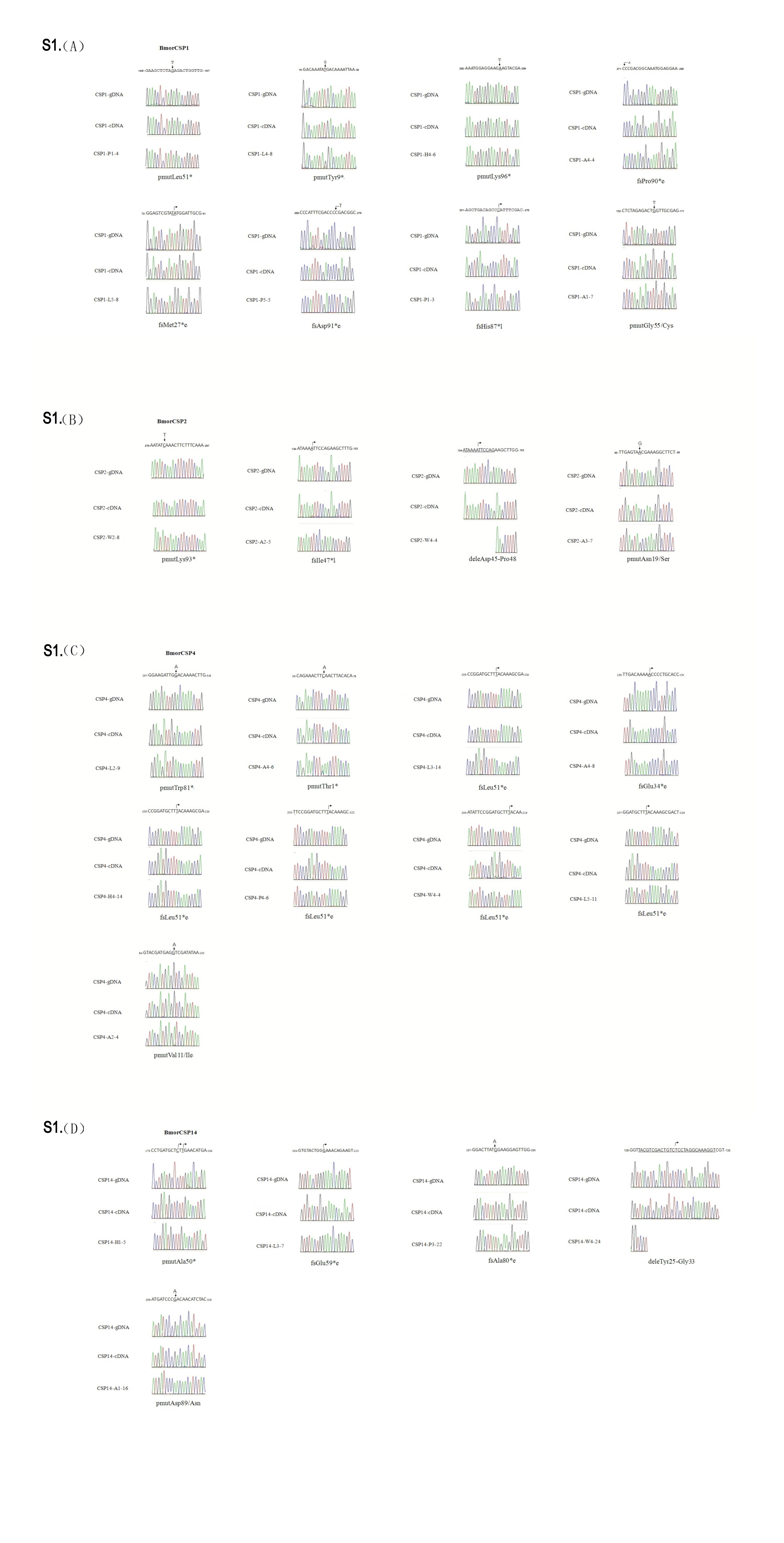

Supplement: Figure S1 — Sequence analysis of gDNA and cDNA PCR products encoding CSPs in various tissues of five individual B. mori females (F1–5). The nucleotide sequences from genomic DNA (gDNA) of a single individual female are shown atop cDNA sequences without RDD from the same single individual female. Specific tissue/individual RDDs are shown below. Only a few examples of RDDs on cDNAs of RNA encoding BmorCSP1 (A), BmorCSP2 (B), BmorCSP4 (C) and BmorCSP14 (D) from the antennae (A4-4, A1–7, A2–5, A3–7, A4–6, A4–8, A2–4 and A1–16), legs (L4–8, L5–8, L2–9, L3–14, L5–11 and L3–7), head (H4–6, H4–14, H1–5), pheromone gland (P1–4, P5-5, P1–3, P4–6, P3–22) and wings (W2–8, W4-4, and W4–24) are represented. Nucleotide insertion, deletion and substitution at the editing sites (RDDs) are underlined. PmutAA: RDD and typo amino acid change, pmutAA*: RDD and change amino acid to stop codon, fsAA*e: Frame-shift RDD and switch to early stop codon position, fsAA*l: Frame-shift RDD and switch to late stop codon position, deleAA: Codon deletion, Pmut: Point mutation (RDD), fs: Frame-shift, *: Stop codon, e: early, l: late. Analyzing twenty cDNA sequences for each individual tissue sample, point mutations (RDDs) are found for all the four BmorCSP genes investigated in this study. No fsAA*e RDDs are found for BmorCSP2. No fsAA*l RDDs are found for BmorCSP4 and BmorCSP14. No deleAA RDDs are found for BmorCSP1 and BmorCSP4. (TIF) [file pone.0086932.s001.tif]

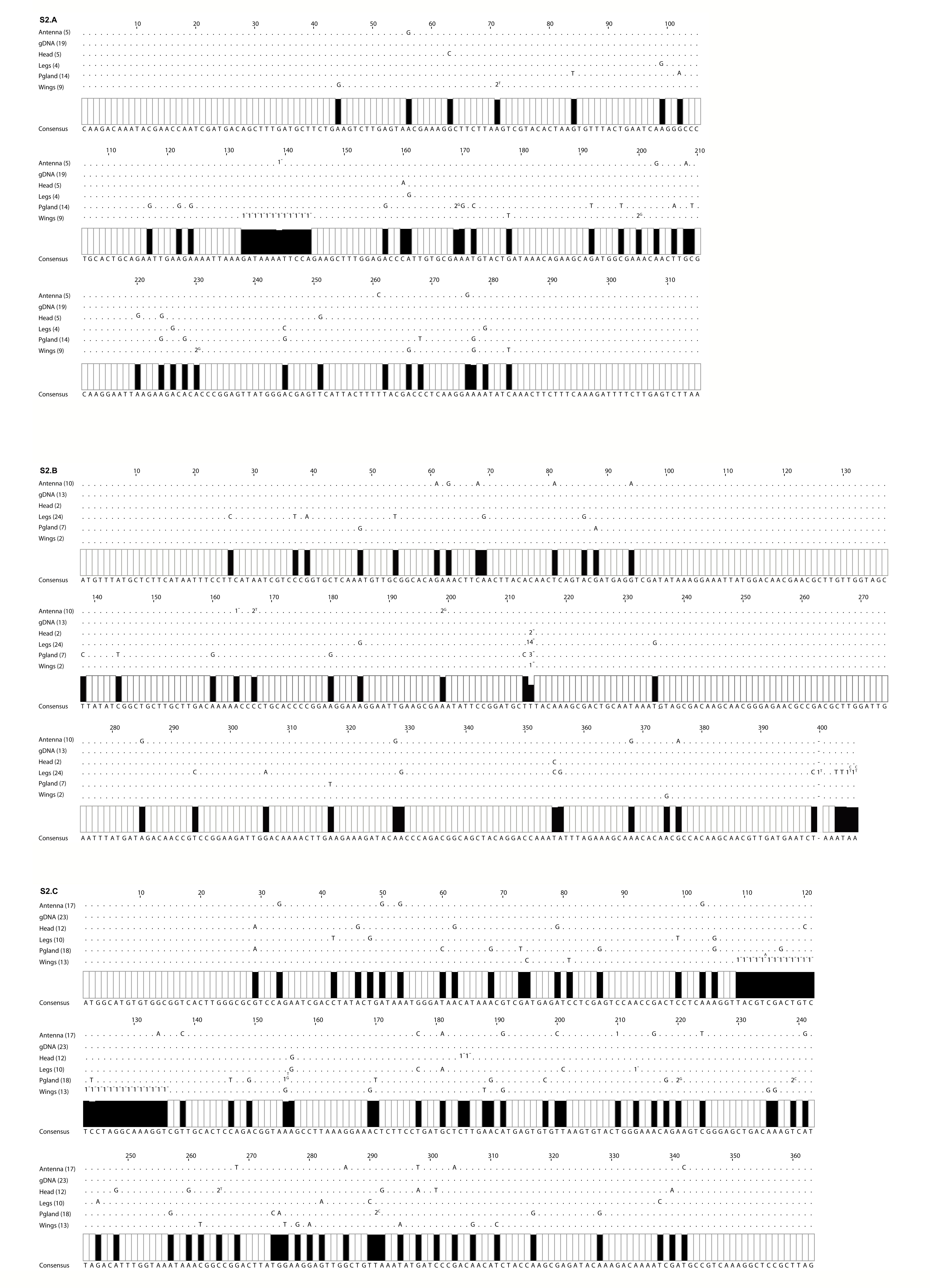

Supplement: Figure S2 — Tissue-specific editing on cDNA of BmorCSP2, BmorCSP4 and BmorCSP14 mRNAs. Sequence analysis of cDNA PCR products encoding BmorCSP2 (A.), BmorCSP4 (B.) and BmorCSP14 (C.) reveals nucleotide insertion, deletion and substitution at the editing sites (black rectangles) in the antennae, head, legs, pheromone gland (Pgland) and wings. The size of the black rectangle is proportional to the frequency of RDDs at this location. The consensus sequence below the alignment corresponds to the nucleotide composition of the genomic DNA sequence (gDNA) encoding BmorCSP2 (A.), BmorCSP4 (B.) and BmorCSP14 (C.). The number in brackets next to tissue indicates the number of CSP clones obtained for each tissue cDNA and gDNA. “.” indicates that the base is similar to the consensus sequence on this location. “A”, “T”, “G”, “C” point out a switch to adenosine, thymidine, guanosine and cytosine base in tissue-specific cDNA sequences, respectively. “1−” indicates base deletion in one sequence of the tissue group. “nA” indicates switch to A in n sequences of the tissue group. Number of mismatches seen in only one tissue: 31–62. Number of mismatches seen in two tissues: 1–7. (TIF) [file pone.0086932.s002.tif]

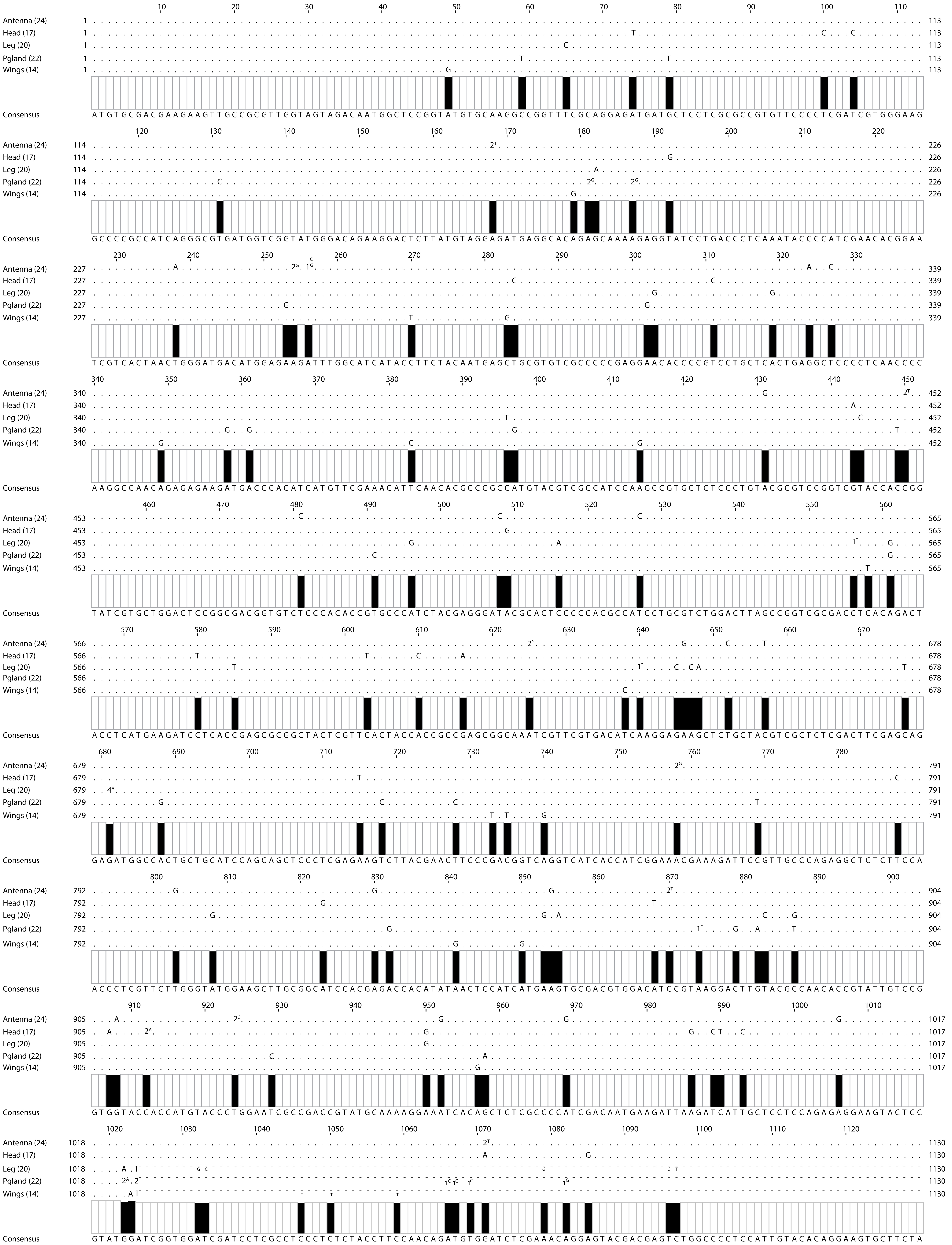

Supplement: Figure S3 — Tissue-specific editing on cDNA of Actin4 mRNAs. Number of mismatches seen in only one tissue: 92. Number of mismatches seen in two tissues: 1. (TIF) [file pone.0086932.s003.tif]

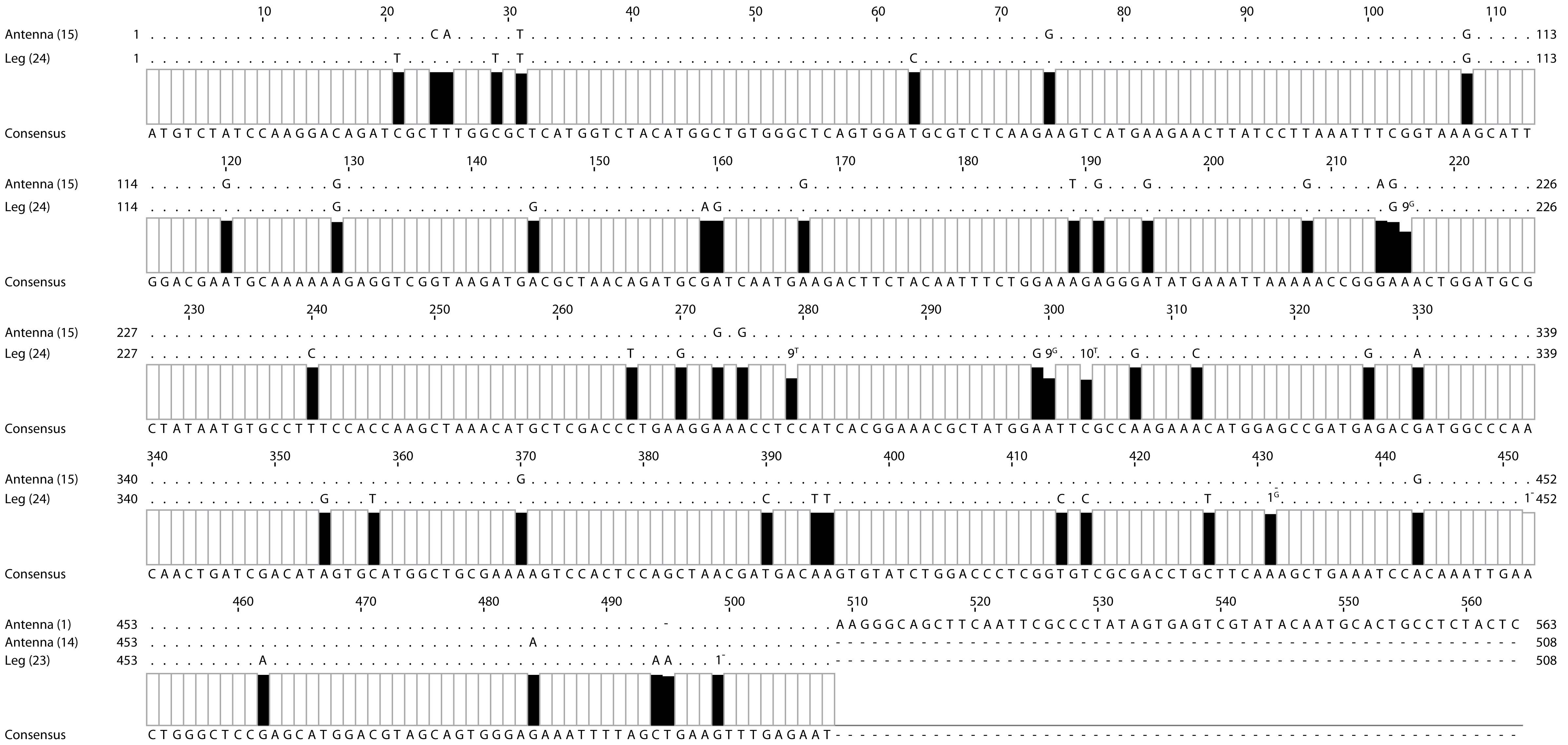

Supplement: Figure S4 — Tissue-specific editing on cDNA of PBP-1 mRNAs. Number of mismatches seen in only one tissue: 39. Number of mismatches seen in two tissues: 3. (TIF) [file pone.0086932.s004.tif]

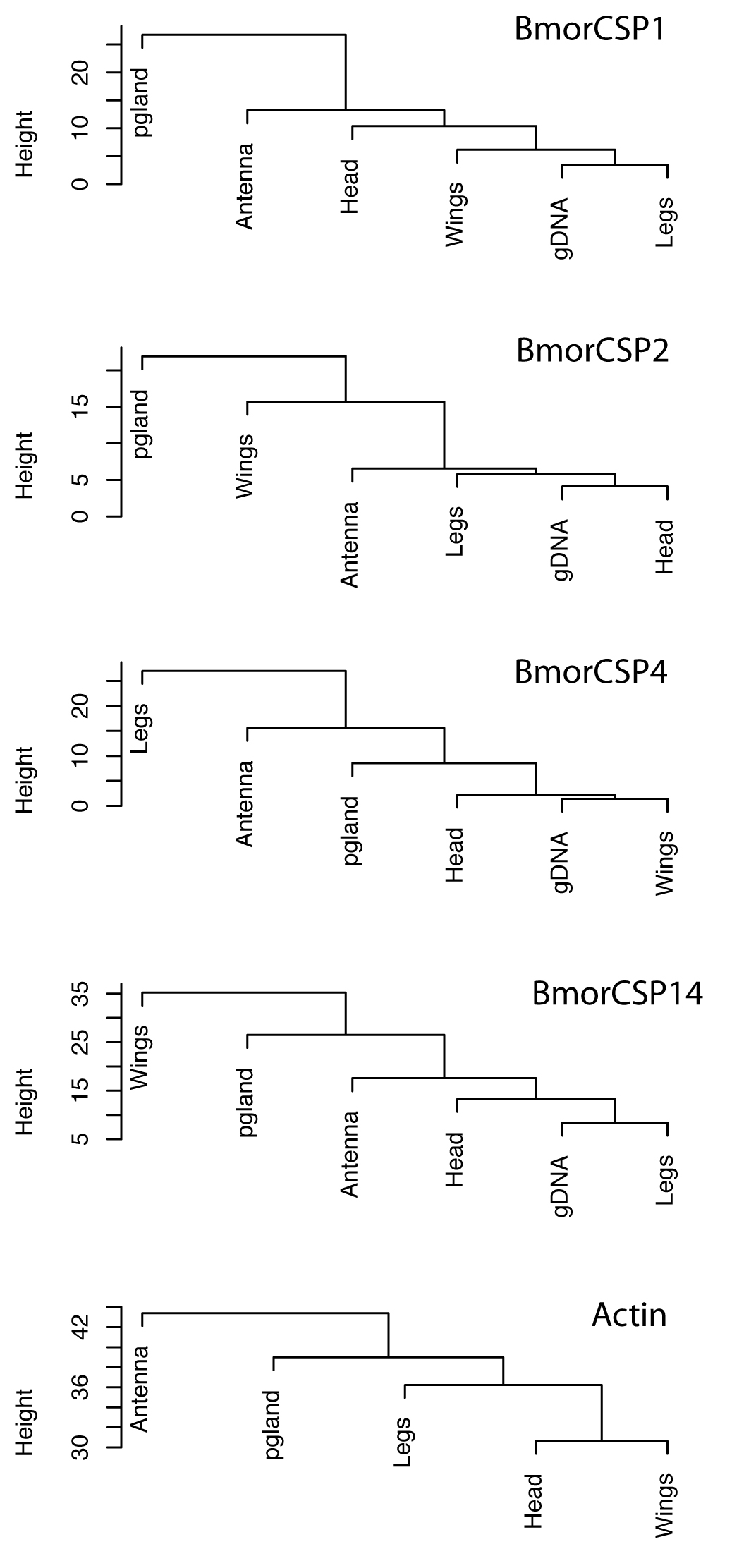

Supplement: Figure S5 — Hierarchical clustering of tissues based on RDDs in BmorCSP and Actin genes (hclust command in R). The distance (height) indicates the number of RDDs in a given tissue. (TIF) [file pone.0086932.s005.tif]

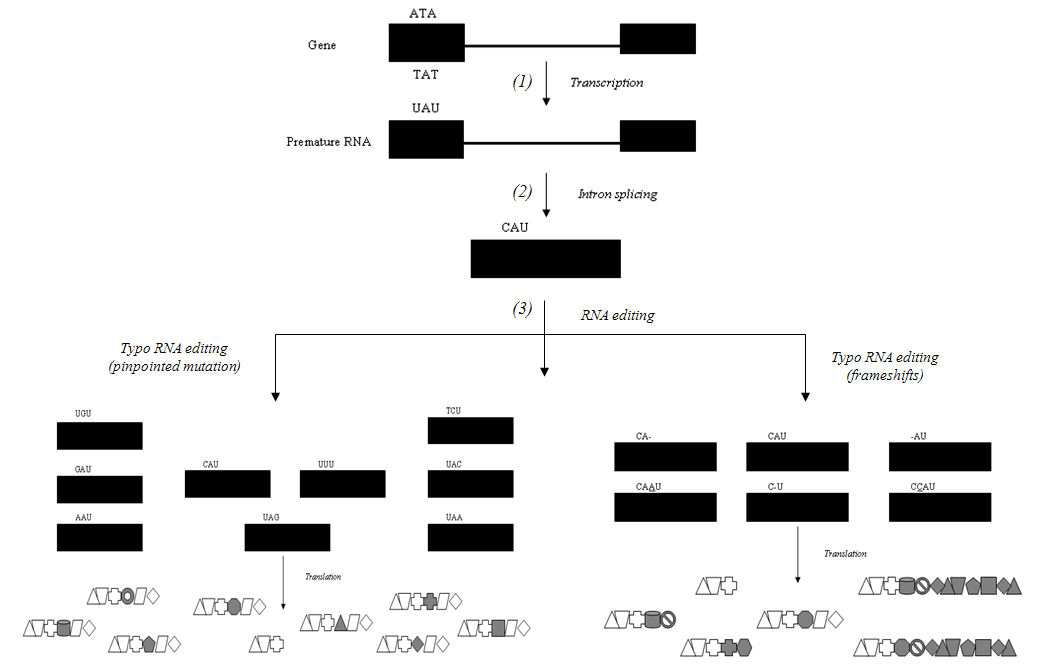

Supplement: Figure S6 — Mutation mechanism of CSPs. Genomic DNA is transcribed into premature mRNA (1) that is processed for intron splicing and excision of non-coding region (2). The mature mRNA is then subjected to typo editing (3). This results in a switch of one or few nucleotides from which all various combinations are possible. One pinpointed mutation (left) in the codon for Tyrosine (UAU) on mRNA can be silent (UAC also codes for Tyr) or can replace Tyr by Asp (GAU), Asn (AAU), His (CAU), Cys (UGU), Phe (UUU), Ser (UCU) and Tyr UAC (Tyr). The substitution of U to A can lead to stop codon and abort CSP. Single base insertions or deletions (right) can induce a shift in the reading frame (frame-shift mutations) resulting in a drastic change in the CSP. The protein can be aborted or have a prominent extra C-terminal tail. Deletions that remove a few juxtaposed bases in the internal part of the CSP-RNA can produce shorter proteins lacking of specific motifs. Consequently, a high number of protein variants are produced from one single CSP gene, rejecting the dogmatic concept ‘One gene-One protein’. (TIF) [file pone.0086932.s006.tif]

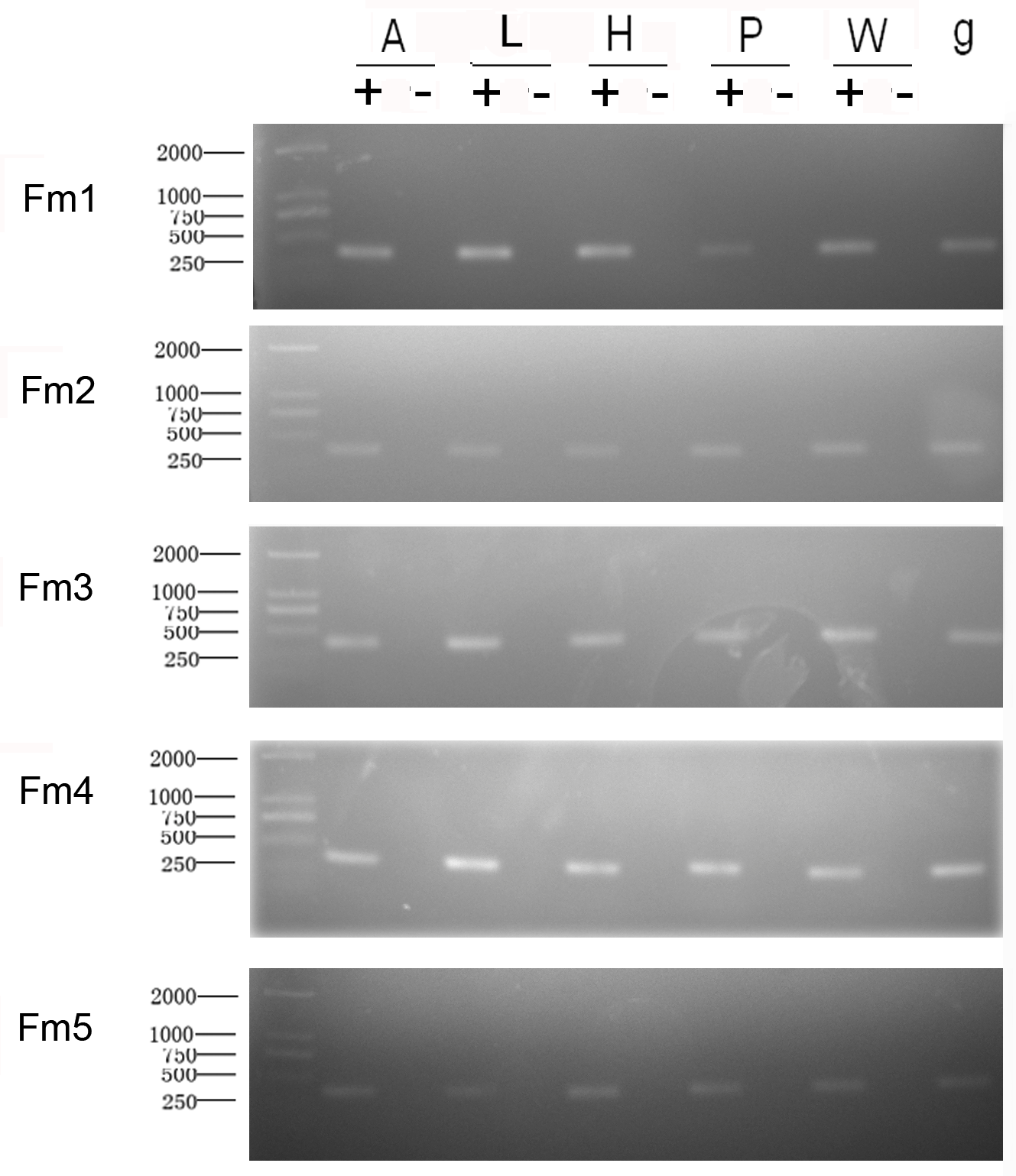

Supplement: Figure S7 — Control experiments on BmorCSP14. Agarose gel electrophoresis of BmorCSP14 encoding cDNA PCR products from the antennae (A), legs (L), head (H), pheromone gland (P) and wings (W) from five individual newly-emerged virgin females of the silkworm moth, B. mori (Fm1, Fm2, Fm3, Fm4 and Fm5). +: RT reaction including the reverse transcriptase, −: RT reaction lacking the reverse transcriptase. No product was amplified in the RT reaction lacking the reverse transcriptase, demonstrating that there is no genomic DNA contamination in RNA samples. (TIF) [file pone.0086932.s007.tif]
