# Supplementary material for: Molecular Evidence of RNA Editing in Bombyx Chemosensory Protein Family
Source: PLoS One. 2014 Feb 13;9(2):e86932. doi: 10.1371/journal.pone.0086932 (PMC3923736; doi:10.1371/journal.pone.0086932)
Supplement: Table S1 — RDDs on cDNA of CSP-RNAs. Pmut: Point mutation, Fs: Frame-shift. pmutAA*: Changes an amino acid to a stop codon and induces shortened protein (lack of the C-terminus). fsAA*e: Leads to amino acid change and modification of stop codon position (early-stop codon). fsAA*l: Leads to amino acid change and modification of stop codon position (late-stop codon). deleAA: Deletes multiple codons and induces shortened protein (lack of central amino acid motifs). A: Antennae, L: Legs, Hd: Head, PG: Pheromone gland, Wg: Wings. F1–5: Individual female 1–5. +: RDDs in the same tissue in different individuals; ++: RDDs in various tissues from the same individual; +++: RDDs in various tissues from different individuals. RDDs on functional elements are bolded and underlined. §: F-L4 T>G on site 72 corresponds to RDD on the signal peptide. Sequencing more CSP14 clones from the (+) reaction identified even more RDDs (point mutations in italic). (DOC) [file pone.0086932.s008.doc]

| **Gene** | **Seq No.** | **Tissue** | **RDD** | **Site** | **AA Change** | **GenBank No.** |
| --- | --- | --- | --- | --- | --- | --- |
| **CSP1** | **7** | **F-A1** | **G>T** | **208** | **Gly to Cys** | JQ085851 |
|  | 5 |  | G>A | 254 | Ser to Asn | JQ085850 |
|  | 7 |  | A>G | 331 | Lys to Glu | JQ085851 |
|  | 6 | F-L1 | G>T | 110 | Arg to Met | JQ085852 |
|  | **1**  **2** | **F-H1** | **C>T** | **349** | **Arg to Cys** | JQ085853  JQ085854 |
|  | 2 | F-P1 | C>G | 112 | Leu to Val | JQ085855 |
|  | 2 |  | G>T | 129 | Met to Ile | JQ085855 |
|  | 1  4  5  7 |  | G>T | 166 | Gly to Trp | JQ085856  JQ085857  JQ085858  JQ253592 |
|  | 1  4 |  | G>T | 202 | pmutAA* | JQ085856  JQ085857 |
|  | 9 |  | T>C | 281 | Leu to Pro | JQ253593 |
|  | 3 |  | C< | 307 | fsAA*l | JQ253594 |
|  | 9 |  | A>G | 355 | Lys to Glu | JQ253593 |
|  | 10 | F-W1 | A>G | 331 | Arg to Gly | JQ253595 |
|  |  | F-A2 | No mutation |  |  |  |
|  |  | F-L2 | No mutation |  |  |  |
|  |  | F-H2 | No mutation |  |  |  |
|  | 5 | F-P2 | G>T | 147 | Lys to Asn | JQ253596 |
|  |  | F-W2 | No mutation |  |  |  |
|  | 3 | F-A3 | G>A | 244 | Ala to Thr | JQ253597 |
|  | 1 |  | T>A | 261 | Asp to Glu | JQ253598 |
|  | 4 | F-L3 | T>C | 113 | Leu to Pro | JQ253599 |
|  | 7 |  | T>C | 210 | Gly | JQ253600 |
|  | 4 |  | A>G | 260 | Asp to Gly | JQ253599 |
|  | 3 | F-H3 | C>T | 150 | Gly | JQ253602 |
|  | 1 |  | C>T | 162 | Pro | JQ253601 |
|  | 1 |  | A>G | 201 | Leu | JQ253601 |
|  | **2** | **F-P3** | **T>C** | **220** | **Cys to Arg** | JQ253603 |
|  | 3 |  | G>A | 235 | Glu to Lys | JQ253604 |
|  | 8 |  | A>G | 278 | Glu to Gly | JQ253605 |
|  | 7 |  | T>C | 288 | Ile | JQ253606 |
|  | 1 |  | C>T | 318 | Pro | JQ253607 |
|  | 5 | F-W3 | A>G | 239 | Lys to Arg | JQ253608 |
|  | 4 |  | T>C | 311 | Phe to Ser | JQ253609 |
|  | 4 |  | G>A | 323 | Gly to Asp | JQ253609 |
|  | **3** | **F-A4** | **G>A** | **155** | **Cys to Tyr** | JQ253610 |
|  | 4 |  | A> | 317 | fsAA*e | JQ253611 |
|  | 8 | F-L4 | T>G | 72 | fsAA*e | § |
|  | 4 | F-H4 | C>A | 162 | Pro | JQ253612 |
|  | 1 |  | A>G | 228 | Glu | JQ253613 |
|  | 6 |  | A>T | 337 | pmutAA* | JQ253614 |
|  | 5 | F-P4 | A>G | 89 | Gln to Arg | JQ253615 |
|  | 1 |  | A>G | 152 | Lys to Arg | JQ253616 |
|  | 6 |  | G>T | 166 | Gly to Trp | JQ253617 |
|  | 5 |  | T>C | 188 | Leu to Pro | JQ253615 |
|  | 7 |  | A>G | 219 | Lys | JQ253618 |
|  | 5 |  | G>A | 244 | Ala to Thr | JQ253615 |
|  | 5 |  | A>G | 279 | Glu | JQ253615 |
|  | 6 |  | G>A | 304 | Ala to Thr | JQ253617 |
|  |  | F-Wg4 | No mutation |  |  |  |
|  | 3 | F-A5 | A>G | 90 | Asn | JQ253619 |
|  | 6 |  | T>C | 113 | Leu to Pro | JQ253620 |
|  | **5** |  | **A>G** | **125** | **Tyr to Cys** | JQ253621 |
|  | 1 |  | T>A | 159 | Thr | JQ253622 |
|  | 5 |  | T>C | 176 | Leu to Pro | JQ253621 |
|  | 1 |  | T>C | 255 | Ser | JQ253622 |
|  | 2 |  | T>C | 281 | Leu to Pro | JQ253623 |
|  | 8 | F-L5 | TA< | 125, 126 | fsAA*e | JQ253624 |
|  | 9 | F-H5 | A>G^++^ | 119 | Glu to Gly | JQ253625 |
|  | 5 |  | A>T | 237 | Glu to Asp | JQ253626 |
|  | 5 | F-P5 | A>G | 103 | Asn to Asp | JQ253627 |
|  | 7 |  | A>G^++^ | 119 | Glu to Gly | JQ253628 |
|  | 5 |  | G>T | 147 | Lys to Asn | JQ253627 |
|  | 7 |  | A>G | 296 | Glu to Gly | JQ253628 |
|  | 5 |  | T> | 318 | fsAA*e | JQ253627 |
|  | 10 |  | G>T | 359 | Ala to Ser | JQ253629 |
|  | 2 | F-W5 | T>C | 189 | Leu | JQ253630 |
| **CSP2** |  | F-A1 | No mutation |  |  |  |
|  |  | F-L1 | No mutation |  |  |  |
|  | 7 | F-H1 | G>C | 111 | Arg to Ser | JQ253640 |
|  | 8 | F-P1 | G>T | 132 | Lys to Asn | JQ253645 |
|  | 3 |  | A>G | 165 | Glu | JQ253646 |
|  | 1 |  | A>G | 170 | Lys to Arg | JQ253647 |
|  | 4 |  | A>G | 218 | Lys to Arg | JQ253648 |
|  | 6 |  | G>T | 240 | Gln to His | JQ253649 |
|  | 8 |  | C>T | 245 | Ala to Val | JQ253645 |
|  | 4 |  | G>A | 254 | Leu to His | JQ253648 |
|  | 2 |  | A>G | 276 | Thr | JQ253650 |
|  | 4 |  | A>G | 293 | Asp to Gly | JQ253648 |
|  | 1 |  | C>T | 316 | Pro to Ser | JQ253647 |
|  | 3 |  | A>G^+++^ | 325 | Lys to Glu | JQ253646 |
|  |  | F-W1 | No mutation |  |  |  |
|  | 5 | F-A2 | A< | 187 | fsAA*l | JQ253631 |
|  | 7 | F-L2 | A>G | 147 | Asn | JQ253636 |
|  |  | F-H2 | No mutation |  |  |  |
|  | 1 | F-P2 | G>A | 150 | Gly | JQ253651 |
|  | 4 |  | A>G | 217 | Lys to Glu | JQ253652 |
|  | 3 | F-W2 | A>G | 92 | Glu to Gly | JQ253659 |
|  | 8 |  | A>G | 314 | Asp to Gly | JQ253660 |
|  | 8 |  | A>G^+++^ | 325 | Lys to Glu | JQ253660 |
|  | 8 |  | C>T | 331 | pmutAA* | JQ253660 |
|  | 7 | F-A3 | A>G | 104 | Asn to Ser | JQ253632 |
|  | 7 |  | G>A | 256 | Ala to Thr | JQ253632 |
|  | 10 |  | A>G | 324 | Gly | JQ253633 |
|  |  | F-L3 | No mutation |  |  |  |
|  | 10 | F-H3 | C>A | 208 | His to Asn | JQ253642 |
|  | 3 |  | A>G | 268 | Lys to Glu | JQ253641 |
|  | 6 | F-P3 | A>G | 172 | Lys to Glu | JQ253655 |
|  | 4 |  | A>G | 217 | Lys to Glu | JQ253654 |
|  | 2 |  | T>C | 220 | Cys to Arg | JQ253653 |
|  | 4 | F-W3 | G>T | 226 | Asp to Tyr | JQ253661 |
|  | 3 | F-A4 | A>G | 251 | Gln to Arg | JQ253634 |
|  | 6 |  | T >C | 310 | Tyr to His | JQ253635 |
|  | 2 | F-L4 | A>G | 274 | Thr to Ala | JQ253637 |
|  | 5 |  | A>C | 293 | Asp to Ala | JQ253638 |
|  | 5 |  | A>G | 327 | Lys | JQ253638 |
|  | 5 | F-H4 | A>G^++^ | 272 | Lys to Arg | JQ253643 |
|  | 1 | F-P4 | A>G | 205 | Thr to Ala | JQ253656 |
|  | 5 |  | A>G^++^ | 272 | Lys to Arg | JQ253657 |
|  | 2  6 | F-W4 | A>T | 119 | Lys to Met | JQ253663  JQ253665 |
|  | 1  4 |  | A---G< | 182-193 | deleAA | JQ253662  JQ253664 |
|  |  | F-A5 | No mutation |  |  |  |
|  | 10 | F-L5 | A>G | 209 | His to Arg | JQ253639 |
|  | **10** | **F-H5** | **T>G** | **299** | **Phe to Cys** | JQ253644 |
|  | 2 | F-P5 | C>T | 257 | Ala to Val | JQ253658 |
|  | 3  5 | F-W5 | A>G | 278 | His to Arg | JQ253666  JQ253667 |
| **CSP4** | 2  7 | F-A1 | C>T | 167 | Pro to Leu | JQ253668  JQ253669 |
|  | 2  7 |  | A>G | 199 | Asn to Asp | JQ253668  JQ253669 |
|  | 10 | F-L1 | T>C | 26 | Phe to Ser | JQ253681 |
|  | 3 |  | C>T | 37 | Phe to Ser | JQ253678 |
|  | 4 |  | C>T | 54 | Cys | JQ253679 |
|  | **10** |  | **A>G** | **86** | **Tyr to Cys** | JQ253681 |
|  | 7 |  | A>G | 329 | Asn to Ser | JQ253680 |
|  | 4 | F-A2 | G>A | 94 | Val to Ile | JQ253670 |
|  | 5 |  | G>A | 376 | Ala to Thr | JQ253671 |
|  | 2 | F-L2 | T>C | 138 | Ala | JQ253682 |
|  | 9 |  | G>A | 306 | fsAA*e | JQ253683 |
|  | 2 | F-A3 | G>A | 61 | Glu to Lys | JQ253672 |
|  | 2  5  7  12  13  14  15 | F-L3 | T< ^+++^ | 215 | fsAA*e | JQ253684  JQ253685  JQ253686  JQ253687  JQ253688  JQ253689  JQ253690 |
|  | 6 | F-A4 | C>A | 68 | pmutAA* | JQ253673 |
|  | 7 |  | T>A | 81 | Thr | JQ253674 |
|  | 8 |  | A< | 164 | fsAA*e | JQ253675 |
|  | 6 |  | A>G | 285 | Ile to Met | JQ253673 |
|  | 9 |  | A>G | 328 | Asn to Asp | JQ253676 |
|  | 7 | F-L4 | A>G | 69 | Ser | JQ253691 |
|  | 6 |  | A>G | 185 | Lys to Arg | JQ253692 |
|  | **4** |  | **A>G** | **356** | **Tyr to Cys** | JQ253693 |
|  | 14  19 | F-H4 | T< ^+++^ | 215 | fsAA*e | JQ253701  JQ253702 |
|  | 4 | F-P4 | A>G | 48 | Lys | JQ253703 |
|  | 7 |  | G>A | 88 | Asp to Asn | JQ253704 |
|  | 4 |  | C>T | 144 | Ile | JQ253703 |
|  | 2 |  | A>G | 160 | Lys to Glu | JQ253705 |
|  | 5 |  | A>G | 180 | Glu | JQ253706 |
|  | 1  6  8 |  | T< ^+++^ | 215 | fsAA*e | JQ253707  JQ253708  JQ253709 |
|  | 8 |  | G>A | 251 | Arg to Gln | JQ253709 |
|  | 8 |  | G>T | 286 | Asp to Tyr | JQ253709 |
|  | 2 |  | A>T | 317 | Glu to Val | JQ253705 |
|  | 4 | F-W4 | T< ^+++^ | 215 | fsAA*e | JQ253711 |
|  | 2 |  | A>G | 374 | Asn to Ser | JQ253710 |
|  | 7 | F-A5 | A>G | 63 | Glu | JQ253677 |
|  | 7 |  | A>G | 368 | Lys to Arg | JQ253677 |
|  | 11 | F-L5 | G>A | 39 | Pro | JQ253694 |
|  | 11  12  5  21  22  23  26 |  | T< ^+++^ | 215 | fsAA*e | JQ253694  JQ253695  JQ253696  JQ253697  JQ253698  JQ253699  JQ253700 |
|  | 22 |  | T>C | 293 | Val to Ala | JQ253698 |
| **CSP14** | 14 | F-A1 | G>T | 268 | Asp to Tyr | JQ253713 |
|  | 16 |  | G>A | 304 | Asp to Asn | JQ253712 |
|  | *23* |  | *A>G* | *35* | *Glu to Gly* | KC879973 |
|  | *22* |  | *T>C* | *101* | *Leu to Pro* | KC879972 |
|  | *24* |  | *A>C* | *163* | *Lys to Gln* | KC879974 |
|  | 14 | F-L1 | A>G | 105 | Lys | JQ253729 |
|  | *22* |  | *C>T* | *28* | *Arg to Cys* | KC879976 |
|  | *18* |  | *G>T* | *49* | *Asp to Tyr* | KC879975 |
|  | *22* |  | *A>G* | *147* | *Pro* | KC879976 |
|  | 5 | F-H1 | C,T < | 184, 186 | pmutAA* | JQ253739 |
|  | *21* |  | *A>G* | *44* | *Tyr to Cys* | KC879977 |
|  | *14* |  | *T>C* | *88* | *Tyr to Cys* | KC879978 |
|  | *17* |  | *A>G* | *269* | *Asp to Gly* | KC879980 |
|  | *12* |  | *A>G* | *278* | *Lys to Arg* | KC879979 |
|  | 1 | F-P1 | A>G | 86 | Glu to Gly | JQ253751 |
|  | 10  15 |  | C>T | 123 | Leu | JQ253755  JQ253756 |
|  | 10  15 |  | C>T | 170 | Thr to Ile | JQ253755  JQ253756 |
|  | 5 |  | A>G | 155 | Lys to Arg | JQ253754 |
|  | 3 |  | A>G | 189 | Glu | JQ253752 |
|  | 10  15 |  | A>G | 220 | Lys to Glu | JQ253755  JQ253756 |
|  | 4 |  | A>G | 317 | Gln to Arg | JQ253753 |
|  | 11 | F-W1 | A>G | 169 | Thr to Ala | JQ253769 |
|  | **11** |  | **G>T** | **276** | **Trp to Cys** | JQ253769 |
|  | 11 |  | T>C | 311 | Ile to Thr | JQ253769 |
|  | *22* |  | *G>A* | *151* | *Gly to Ser* | KC879982 |
|  | *21* |  | *A>G* | *227* | *Lys to Glu* | KC879981 |
|  | 11 | F-A2 | A>G | 33 | Pro | JQ253716 |
|  | 4 |  | G>A ^++^ | 181 | Ala to Thr | JQ253714 |
|  | 4 |  | T>C | 200 | Val to Ala | JQ253714 |
|  | 7 |  | A>G | 216 | Lys | JQ253717 |
|  | 8 |  | T>C | 342 | Asp | JQ253715 |
|  | *17* |  | *A>G* | *62* | *Asn to Ser* | KC879983 |
|  | *22* |  | *A>G* | *237* | *Lys* | KC879984 |
|  | 3 | F-L2 | C>T | 42 | Thr | JQ253730 |
|  | 3 |  | C>T | 99 | Leu | JQ253730 |
|  | 7 |  | G>A ^++^ | 181 | Ala to Thr | JQ253731 |
|  | 10 |  | G>A | 282 | Glu | JQ253732 |
|  | *22* |  | *T>C* | *48* | *Thr* | KC879985 |
|  | *22* |  | *T>C* | *101* | *Leu to Pro* | KC879985 |
|  | *13* |  | *T>C* | *200* | *Val to Ala* | KC879986 |
|  | *14* |  | *G>A* | *213* | *Gly* | KC879987 |
|  | 5 | F-H2 | C>T | 184 | Leu to Phe | JQ253740 |
|  | *18* |  | *G>A* | *195* | *Glu* | KC879988 |
|  | *22* |  | *A>G* | *241* | *Ile to Val* | KC879989 |
|  | *22* |  | *G>A* | *304* | *Asp to Asn* | KC879989 |
|  | *16* |  | *A>G* | *326* | *Tyr to Cys* | KC879990 |
|  | 1 | F-P2 | T>C | 60 | Asp | JQ253757 |
|  | 6  11 |  | T>C | 239 | Val to Ala | JQ253758  JQ253759 |
|  | 6  11 |  | T>C | 291 | Val | JQ253758  JQ253759 |
|  | *19* |  | *G>A* | *163* | *Arg to Gln* | KC879991 |
|  | *18* |  | *A>G* | *293* | *Lys to Arg* | KC879992 |
|  | *15* |  | *T>C* | *300* | *Asp* | KC879993 |
|  | *21* |  | *G>A* | *319* | *Ala to Thr* | KC879994 |
|  | *23* |  | *A>G* | *324* | *Arg* | KC879995 |
|  | *21* | F-W2 | *A>T* | *116* | *Asp to Val* | KC879997 |
|  | *20* |  | *A>G* | *169* | *Thr to Ala* | KC879996 |
|  | 16 | F-A3 | A>G | 50 | Asp to Gly | JQ253718 |
|  | 16 |  | A>G | 53 | Lys to Arg | JQ253718 |
|  | 14 |  | G>A | 134 | Gly to Asp | JQ253724 |
|  | 4 |  | T>C | 138 | Arg | JQ253722 |
|  | 3 |  | T>C | 210 | Thr | JQ253721 |
|  | 21 |  | C>T | 224 | Ser to Leu | JQ253720 |
|  | 13 |  | A>G | 241 | Ile to Val | JQ253723 |
|  | 12 |  | G>T | 298 | Asp to Tyr | JQ253719 |
|  | 3 |  | T>C | 313 | Tyr to His | JQ253721 |
|  | *30* |  | *T>C* | *271* | *Leu* | KC879998 |
|  | 13 | F-L3 | T>G | 48 | Thr | JQ253733 |
|  | 13 |  | G>A | 245 | Arg to Lys | JQ253733 |
|  | 7 |  | G< | 213 | fsAA*e | JQ253734 |
|  | *20* |  | *T>C* | *118* | *Cys to Arg* | KC879999 |
|  | *11* |  | *T>C* | *122* | *Leu to Pro* | KC880000 |
|  | 21 | F-H3 | A>G | 46 | Thr to Ala | JQ253742 |
|  | 2 |  | A>G | 62 | Asn to Ser | JQ253741 |
|  | 21 |  | A>G | 79 | Ile to Val | JQ253742 |
|  | 2 |  | C>T | 265 | Pro to Ser | JQ253741 |
|  | 22 |  | A>G | 292 | Lys to Glu | JQ253743 |
|  | *18* |  | *A>G* | *77* | *Glu to Gly* | KC880001 |
|  | *18* |  | *A>G* | *221* | *Lys to Arg* | KC880001 |
|  | 6 | F-P3 | G>A | 29 | Arg to His | JQ253760 |
|  | 3 |  | A>G | 68 | Asn to Ser | JQ253761 |
|  | 2 |  | A>G | 105 | Lys | JQ253762 |
|  | 22 |  | A>T | 155 | Lys to Ile | JQ253763 |
|  | 2 |  | T>C | 198 | Cys | JQ253762 |
|  | 22 |  | A>G | 218 | Gln to Arg | JQ253763 |
|  | 22 |  | G>A | 275 | fsAA*e | JQ253763 |
|  | *17* |  | *C>T* | *28* | *Arg to Cys* | KC880003 |
|  | *13* |  | *C>T* | *209* | *Thr to Ile* | KC880002 |
|  | *17* |  | *A>G* | *228* | *Gly* | KC880003 |
|  | 7 | F-W3 | A>C | 74 | Asp to Ala | JQ253770 |
|  | 17 |  | T>A | 113 | Val to Asp | JQ253771 |
|  | 3 |  | A>G | 236 | Lys to Arg | JQ253772 |
|  | *21* |  | *A>C* | *53* | *Lys to Thr* | KC880004 |
|  | *15* |  | *C>T* | *136* | *Arg to Cys* | KC880005 |
|  | 8 | F-A4 | A>G | 103 | Lys to Glu | JQ253725 |
|  | *18* |  | *A>G* | *162* | *Leu* | KC880006 |
|  | 14 | F-L4 | A>G | 156 | Lys | JQ253737 |
|  | 14 |  | T>C | 177 | Pro | JQ253737 |
|  | 1 |  | T>C | 290 | Val to Ala | JQ253735 |
|  | 11 |  | T>C | 338 | Ile to Thr | JQ253736 |
|  | *22* |  | *T>C* | *109* | *Tyr to His* | KC880008 |
|  | *15* |  | *C>T* | *312* | *Ile* | KC880007 |
|  | 1 | F-H4 | G>A | 156 | Lys | JQ253744 |
|  | 22 |  | A>G | 248 | His to Arg | JQ253745 |
|  | 6 |  | A>G | 260 | Lys to Arg | JQ253747 |
|  | 22 |  | C>T | 265 | Pro to Ser | JQ253745 |
|  | 11 |  | G>A | 298 | Asp to Asn | JQ253746 |
|  | 2 |  | C>T | 301 | Pro to Ser | JQ253748 |
|  | 11 |  | G>A | 340 | Asp to Asn | JQ253746 |
|  | *20* |  | *G>T* | *137* | *Arg to Leu* | KC880009 |
|  | *23* |  | *A>G* | *235* | *Lys to Glu* | KC880010 |
|  | *19* |  | *A>G* | *322* | *Arg to Gly* | KC880011 |
|  | *19* |  | *A>G* | *335* | *Lys to Arg* | KC880011 |
|  | *39* | F-P4 | *A>G* | *74* | *Asp to Gly* | KC880012 |
|  | *17* |  | *A>G* | *215* | *Lys to Arg* | KC880013 |
|  | 14 | F-W4 | C>T | 81 | Ile | JQ253773 |
|  | 24 |  | T---T< | 109-135 | deleAA | JQ253774 |
|  | 3 |  | A>T | 188 | Glu to Val | JQ253775 |
|  | 11 |  | A>G | 191 | His to Arg | JQ253776 |
|  | 12 |  | A>G | 278 | Lys to Arg | JQ253777 |
|  | 3 |  | G>A | 280 | Glu to Lys | JQ253775 |
|  | *13* |  | *C>G* | *26* | *Ala to Gly* | KC880014 |
|  | *18* |  | *T>C* | *30* | *Arg* | KC880015 |
|  | *18* |  | *T>C* | *210* | *Thr* | KC880015 |
|  | *24* |  | *A>G* | *269* | *Asp to Gly* | KC880016 |
|  | 8 | F-A5 | T>C | 177 | Pro | JQ253727 |
|  | 4 |  | A>G | 191 | His to Arg | JQ253728 |
|  | 3 |  | G>A | 286 | Ala to Thr | JQ253726 |
|  | *18* |  | *G>A* | *27* | *Ala* | KC880017 |
|  | *21* |  | *A>G* | *68* | *Asn to Ser* | KC880018 |
|  | 3 | F-L5 | T>C | 201 | Val | JQ253738 |
|  | *14* |  | *A>G* | *189* | *Glu* | KC880019 |
|  | 19 | F-H5 | G>A | 29 | Arg to His | JQ253750 |
|  | 6 |  | T>C | 120 | Cys | JQ253749 |
|  | *22* |  | *T>A* | *288* | *Ala* | KC880020 |
|  | *22* |  | *C>T* | *306* | *Asp* | KC880020 |
|  | 13 | F-P5 | G>T | 73 | Asp to Tyr | JQ253765 |
|  | 4 |  | A>G | 116 | Asp to Gly | JQ253764 |
|  | 3 |  | C>T | 146 | Pro to Leu | JQ253766 |
|  | 13 |  | A>G | 149 | Asp to Gly | JQ253765 |
|  | 12 |  | A>G | 257 | Asn to Ser | JQ253768 |
|  | 4 |  | C>T | 274 | Asn to Arg | JQ253764 |
|  | 12 |  | A>G | 328 | Lys to Glu | JQ253768 |
|  | 1 |  | G>A | 340 | Asp to Asn | JQ253767 |
|  | *20* |  | *T>C* | *185* | *Leu to Pro* | KC880021 |
|  | *16* |  | *G>A* | *323* | *Arg to Lys* | KC880022 |
|  | 6 | F-W5 | A>G | 155 | Lys to Arg | JQ253779 |
|  | 4 |  | A>G | 235 | Lys to Glu | JQ253778 |
|  | 16 |  | C>T | 262 | Arg to Trp | JQ253781 |
|  | 9 |  | T>A | 295 | Tyr to Asn | JQ253780 |
|  | 4 |  | A>G | 307 | Asn to Asp | JQ253778 |
|  | *21* |  | *T>C* | *171* | *Thr* | KC880024 |
|  | *21* |  | *T>C* | *182* | *Ala* | KC880024 |
|  | *24* |  | *G>A* | *195* | *Glu* | KC880025 |
|  | *15* |  | *T>C* | *210* | *Thr* | KC880026 |
|  | *20* |  | *A>G* | *220* | *Lys to Glu* | KC880023 |
