# Supplementary material for: Molecular Evidence of RNA Editing in Bombyx Chemosensory Protein Family
Source: PLoS One. 2014 Feb 13;9(2):e86932. doi: 10.1371/journal.pone.0086932 (PMC3923736; doi:10.1371/journal.pone.0086932)
Supplement: Table S2 — RDDs on cDNA of Actin and PBP-RNAs. Pmut: Point mutation, Fs: Frame-shift. fsAA*e: Leads to amino acid change and modification of stop codon position (early-stop codon). fsAA*l: Leads to amino acid change and modification of stop codon position (late-stop codon). No changes of an amino acid to a stop codon and induction of shortened protein (pmutAA* RDD) and no deleAA RDDs (deletes multiple codons and induces shortened protein lacking of central amino acid motifs) have been detected on Actin. A: Antennae, L: Legs, Hd: Head, PG: Pheromone gland, Wg: Wings. F1–5: Individual female 1–5. +: RDDs in the same tissue in different individuals; ++: RDDs in various tissues from the same individual; +++: RDDs in various tissues from different individuals. (DOC) [file pone.0086932.s009.doc]

| **Gene** | **Seq No.** | **Tissue** | **RDD** | **Site** | **AA Change** | **GenBank No.** |
| --- | --- | --- | --- | --- | --- | --- |
| **ACTIN4** | 5,27 | F-A1 | A>T | 168 | Gly | KC879871  KC879875 |
|  | 33 |  | T>C | 327 | Ala | KC879873 |
|  | 27 |  | T>C | 481 | Ser to Pro | KC879875 |
|  | 27 |  | T>C | 803 | Leu to Trp | KC879875 |
|  | 5 |  | A>G | 830 | Glu to Gly | KC879871 |
|  | 39 |  | G>A | 908 | Gly to Asp | KC879870 |
|  | 6 |  | A>G | 952 | Ile to Val | KC879872 |
|  | 26 |  | A>G | 969 | Pro | KC879874 |
|  | 39 |  | A>G | 1006 | Arg to Gly | KC879870 |
|  | 7 | F-L1 | A>G | 303 | Glu | KC879877 |
|  | 1 |  | C>T | 583 | Thr | KC879876 |
|  | 30 |  | A>C | 647 | Lys to Thr | KC879878 |
|  | 7 |  | G>A | 648 | Lys | KC879877 |
|  | 22 |  | A>G | 808 | Met to Val | KC879879 |
|  | 1 |  | A>G^+++^ | 950 | Glu to Gly | KC879876 |
|  | 1 |  | G>A^+++^ | 1022 | fAA*e | KC879876 |
|  | 16 |  | T>C | 1033 | Ser to Pro | KC879880 |
|  | 5 | F-H1 | A>T | 74 | Asp to Val | KC879883 |
|  | 4 |  | T>C | 284 | Leu to Pro | KC879882 |
|  | 3 |  | A>G | 823 | Ile to Val | KC879881 |
|  | 19 | F-P1 | G>T | 79 | Ala to Ser | KC879884 |
|  | 11 |  | A>G^+^ | 181 | Ser to Gly | KC879885 |
|  | 15 |  | T>C | 718 | Ser to Pro | KC879886 |
|  | 15 |  | G>A | 958 | Ala to Thr | KC879886 |
|  | 18 |  | A>C | 1066 | Met to Leu | KC879887 |
|  | 14 |  | T>C | 1067 | Met to Thr | KC879888 |
|  | 1 | F-W1 | C>T | 270 | Thr | KC879889 |
|  | 1 |  | G>T | 733 | Asp to Tyr | KC879889 |
|  | 12 |  | A>G | 841 | Asn to Asp | KC879891 |
|  | 9 |  | C>T | 1059 | Phe | KC879890 |
|  | 1 |  | G>A^+++^ | 1022 | fAA*e | KC879889 |
|  | 1 | F-A2 | T>A | 238 | Trp to Arg | KC879892 |
|  | 8 |  | G>A | 324 | Glu | KC879893 |
|  | 1 |  | T>C | 652 | Cys to Arg | KC879892 |
|  | 7,2 |  | A>G | 254 | Lys to Arg | KC879894  KC879895 |
|  | 4 |  | A>G^+^ | 256 | Ile to Leu | KC879896 |
|  | 4 |  | A>G | 646 | Lys to Glu | KC879896 |
|  | 5 | F-L2 | T>C | 1096 | Ser to Pro | KC879897 |
|  | 8 |  | C>T | 1097 | Ser to Phe | KC879898 |
|  | 7 | F-H2 | T>C | 311 | Val to Ala | KC879899 |
|  | 11 |  | A>G | 509 | Tyr to Cys | KC879900 |
|  | 3 |  | A>C | 610 | Thr to Pro | KC879901 |
|  | 5 |  | G>A | 616 | Glu to Lys | KC879902 |
|  | 3 |  | T>C | 788 | Phe to Ser | KC879901 |
|  | 13 |  | A>G^+++^ | 950 | Glu to Gly | KC879903 |
|  | 3 |  | T>C | 989 | Ile to Thr | KC879901 |
|  | 7 | F-P2 | T>C | 131 | Val to Ala | KC879904 |
|  | 7 |  | A>G | 253 | Lys to Glu | KC879904 |
|  | 7 |  | T>C | 728 | Lys to Pro | KC879904 |
|  | 7 |  | C>T^+++^ | 887 | Ala to Val | KC879904 |
|  | 2,9 |  | G>A^+++^ | 1022 | fAA*e | KC879905  KC879906 |
|  | 6 | F-W2 | G>A | 588 | Glu | KC879907 |
|  | 27 |  | T>C | 851 | Met to Thr | KC879908 |
|  | 10,15 |  | T>C | 1025 | Ile to Thr | KC879909  KC879910 |
|  | 14 |  | T>C | 508 | Tyr to His | KC879911 |
|  | 10,11 |  | A>G | 625 | Ile to Val | KC879912  KC879913 |
|  | 1 | F-L3 | A>G | 319 | Thr to Ala | KC879914 |
|  | 1 |  | C>T | 396 | Ala | KC879914 |
|  | 3 |  | A>G | 496 | Ile to Val | KC879915 |
|  | 7 |  | G>C | 645 | Glu to Asp | KC879917 |
|  | 5 |  | A>G,G>A | 853,855 | Lys to Glu | KC879916 |
|  | 3 |  | A>G | 1032 | Gly | KC879915 |
|  | 4,5 | F-H3 | C>A | 912 | Thr | KC879919  KC879919 |
|  | 12 |  | A>G | 986 | Lys to Arg | KC879918 |
|  | 12 |  | C>T | 990 | Ile | KC879918 |
|  | 3 | F-P3 | A>G^+^ | 181 | Ser to Gly | KC879923 |
|  | 2,4 |  | A>G | 187 | Arg to Gly | KC879922  KC879924 |
|  | 6 |  | T>C | 491 | Val to Arg | KC879925 |
|  | 1 |  | A>G^+++^ | 561 | Thr | KC879921 |
|  | 3 |  | C>T | 769 | Arg to Cys | KC879923 |
|  | 3 |  | T>C | 1069 | Trp to Arg | KC879923 |
|  | 1 | F-W3 | A>G | 179 | Asn to Arg | KC879926 |
|  | 5 |  | C>T | 558 | Leu | KC879928 |
|  | 17 |  | A>G | 740 | Asn to Arg | KC879930 |
|  | 2 |  | A>G | 850 | Met to Val | KC879927 |
|  | 2 |  | C>T | 1046 | Ser to Phe | KC879927 |
|  | 9 |  | C>T | 1050 | Leu | KC879929 |
|  | 3,6 | F-A4 | C>T | 450 | Thr | KC879931  KC879932 |
|  | 9 |  | C>T | 527 | Ile to Thr | KC879933 |
|  | 3,6 |  | C>T | 870 | Ile | KC879931  KC879932 |
|  | 3,6 |  | T>C | 924 | Pro | KC879931  KC879932 |
|  | 6 | F-L4 | T>C | 444 | Arg | KC879935 |
|  | 22 |  | A>G^+++^ | 561 | Thr | KC879937 |
|  | 22 |  | A< | 640 | fAA*e | KC879937 |
|  | 6 |  | C>T | 676 | fAA*e | KC879935 |
|  | 11 |  | C>G^+++^ | 887 | Ala to Gly | KC879934 |
|  | 16 |  | A>G | 1079 | Lys to Arg | KC879936 |
|  | 11 | F-H4 | T>C | 100 | Ser to Pro | KC879938 |
|  | 11 |  | T>C | 104 | Ile to Thr | KC879938 |
|  | 11 |  | C>T | 580 | Leu to Phe | KC879938 |
|  | 5 |  | A>T | 868 | Ile to Phe | KC879939 |
|  | 8 | F-P4 | C>T | 59 | Ala to Val | KC879943 |
|  | 2 |  | A>G | 358 | Met to Val | KC879941 |
|  | 8 |  | A>G | 361 | Thr to Ala | KC879943 |
|  | 9 |  | A>G | 397 | Met to Val | KC879944 |
|  | 8 |  | C>T | 449 | Thr to Ile | KC879943 |
|  | 7 |  | A< | 874 | fAA*e | KC879942 |
|  | 1 |  | A>G | 1082 | Asn to Arg | KC879940 |
|  | 18 | F-W4 | A>G | 957 | Thr | KC879945 |
|  | 2 | F-A5 | A>G^+^ | 256 | Ile to Val | KC879947 |
|  | 9 |  | A>G | 431 | Tyr to Cys | KC879950 |
|  | 5 |  | C>T | 657 | Tyr | KC879948 |
|  | 16,6 |  | A>G | 758 | Asn to Ser | KC879946  KC879949 |
|  | 2 |  | A>G | 854 | Lys to Arg | KC879947 |
|  | 10,15 |  | G>T^++^ | 1071 | Trp to Cys | KC879951  KC879952 |
|  | 2 | F-L5 | T>C | 65 | Phe to Ser | KC879954 |
|  | 15 |  | G>A | 182 | Ser to Asn | KC879953 |
|  | 6 |  | C>A | 516 | Leu | KC879957 |
|  | 4 |  | C< | 556 | fAA*e | KC879955 |
|  | 3 |  | T>C | 883 | Tyr to His | KC879956 |
|  | 24 | F-H5 | T>G | 192 | Gly | KC879959 |
|  | 33 |  | G>A | 443 | Arg to His | KC879960 |
|  | 33 |  | A>T | 715 | fAA*e | KC879960 |
|  | 34 |  | G>A | 907 | Gly to Ser | KC879958 |
|  | 30 |  | G>A^++^ | 1071 | fAA*e | KC879961 |
|  | 24 |  | A>G | 1085 | Glu to Gly | KC879959 |
|  | 2 | F-P5 | A>G | 688 | Thr to Ala | KC879962 |
|  | 2 |  | G>A | 882 | Leu | KC879962 |
|  | 1 |  | A>G | 302 | Glu to Gly | KC879963 |
|  | 10 |  | A>T | 340 | fAA*e | KC879966 |
|  | 9 |  | A>G | 832 | Thr to Ala | KC879964 |
|  | 1 |  | C>G | 879 | Asp to Glu | KC879963 |
|  | 3 |  | T>C | 929 | Ile to Thr | KC879965 |
|  | 30 | F-W5 | A>G | 49 | Met to Val | KC879971 |
|  | 25 |  | C>G | 283 | Leu to Val | KC879969 |
|  | 27 |  | A>G | 349 | Arg to Gly | KC879970 |
|  | 27 |  | T>G | 383 | Phe to Ser | KC879970 |
|  | 21 |  | A>G | 414 | Asn | KC879968 |
|  | 25 |  | T>C | 638 | Ile to Thr | KC879969 |
|  | 19 |  | T>C | 735 | Asp | KC879967 |
| **PBP1** | 26 | F-A1 | A>G | 74 | Glu to Gly | KC879832 |
|  | 21 |  | G>A | 484 | Glu to Lys | KC879833 |
|  | 14 | F-L1 | A>G | 145 | Thr to Ala | KC879834 |
|  | 24 |  | G>A | 159 | Ala | KC879837 |
|  | 13 |  | A>G | 160 | Ile to Val | KC879838 |
|  | 32 |  | C>T | 266 | Pro to Leu | KC879839 |
|  | 24 |  | A>G | 270 | Glu | KC879837 |
|  | 24 |  | A>G | 299 | Glu to Gly | KC879837 |
|  | 20 |  | A>C | 312 | Lys to Asn | KC879840 |
|  | 16 |  | A>G | 326 | Glu to Gly | KC879835 |
|  | 13 |  | G>A | 330 | Thr | KC879838 |
|  | 23 |  | G>A | 462 | Pro | KC879836 |
|  | 20 | F-A2 | A>T | 189 | Lys to Asn | KC879843 |
|  | 27 |  | A>G | 191 | Glu to Gly | KC879844 |
|  | 12 |  | A>G | 275 | Asn to Ser | KC879841 |
|  | 12 |  | A>G | 370 | Lys to Glu | KC879841 |
|  | 15 |  | A>G | 443 | His to Arg | KC879842 |
|  | 13 | F-L2 | C>T | 29 | Ala to Val | KC879846 |
|  | 13 |  | C>T^+++^ | 31 | Leu to Phe | KC879846 |
|  | 11 |  | T>C | 63 | Asp | KC879845 |
|  | 20 |  | A>G^+++^ | 129 | Lys | KC879847 |
|  | 11 |  | T>C | 240 | Leu | KC879845 |
|  | 16 |  | A>G | 354 | Ile to Met | KC879849 |
|  | 17 |  | C>T | 358 | His to Tyr | KC879850 |
|  | 11 |  | C>T | 426 | Cys | KC879845 |
|  | 22 |  | A< | 432 | fsAA*e | KC879848 |
|  | 1 | F-A3 | A>G | 120 | Glu | KC879851 |
|  | 1 |  | A>G^+++^ | 129 | Lys | KC879851 |
|  | 3 |  | A>G | 208 | Asp to Asn | KC879852 |
|  | 4 | F-L3 | A>G^+++^ | 215 | Glu to Gly | KC879856 |
|  | 1.3.11 |  | A>G | 216 | Gly | KC879853  KC879855  KC879854 |
|  | 1.3.11 |  | C>T | 279 | Leu | KC879853  KC879855  KC879854 |
|  | 1.3.11 |  | A>G | 300 | Glu | KC879853  KC879855  KC879854 |
|  | 1.3.9.11 |  | C>T | 303 | Phe | KC879853  KC879855  KC879857  KC879854 |
|  | 9 |  | T>C | 414 | Gly | KC879857 |
|  | 4 |  | T>C | 416 | Val to Ala | KC879856 |
|  | 15 | F-A4 | T>A | 25 | Leu to Met | KC879860 |
|  | 13 |  | C>T^+++^ | 31 | Leu to Phe | KC879858 |
|  | 15 |  | A>G | 167 | Glu to Gly | KC879860 |
|  | 14 |  | A>G | 195 | Gly | KC879859 |
|  | 12 | F-L4 | C>T | 21 | Ile | KC879861 |
|  | 27 |  | A>G | 307 | Lys to Glu | KC879862 |
|  | 29 |  | T>C | 390 | Asp | KC879863 |
|  | 19 | F-A5 | T>C | 24 | Ala | KC879864 |
|  | 22 |  | A>G^++^ | 108 | Lys | KC879866 |
|  | 20 |  | G>A | 214 | Glu to Lys | KC879865 |
|  | 22 |  | A>G^+++^ | 215 | Glu to Gly | KC879866 |
|  | 19 |  | A>G | 273 | Glu | KC879864 |
|  | 48 | F-L5 | A>G^++^ | 108 | Gly | KC879867 |
|  | 55 |  | AA>TT | 394，395 | Lys to Leu | KC879869 |
|  | 26 |  | A>G | 431 | Lys to Arg | KC879868 |
