# Supplementary material for: Molecular Evidence of RNA Editing in Bombyx Chemosensory Protein Family
Source: PLoS One. 2014 Feb 13;9(2):e86932. doi: 10.1371/journal.pone.0086932 (PMC3923736; doi:10.1371/journal.pone.0086932)
Supplement: Table S3 — P1 de novo peptide sequences. Amino acid replacements are shown in bold and underlined. (DOC) [file pone.0086932.s010.doc]

| **Peptide No.** | **Peptide Sequence** | **Peptide Mass (Da)** | **NCBI (Blastp)** | **Protein Name** | **Gene Acc. No.** | **Identity (%)** |
| --- | --- | --- | --- | --- | --- | --- |
| ***Trypsin*** |  |  |  |  |  |  |
| 3944 | DDKYTDKYDK | 1289.5774 | AFD97756 | BmorCSP1 | BGIBMGA004045 | 100 |
| 6425 | KCTE**DK** | 722.3268 | AFF18017 |  |  | 67 |
| 6902 | DHLQEALETGC**G**EK | 1528.6826 | AFF18017 |  |  | 93 |
| 6922 | ELTAHFDPDGK | 1228.5723 | AFF18017 |  |  | 100 |
| 8008 | ELKDHLQEALETG**G**CEK | 1898.9043 | AFF18017 |  |  | 94 |
| 10050 | NELELWK | 930.4811 | AFF18017 |  |  | 100 |
| 10602 | LNLQELLENKR | 1368.7725 | AFD97756 |  |  | 100 |
| 10790 | **Q**ETSLDYLLK | 1208.6289 | AFF18017 |  |  | 90 |
| 12598 | LNLQELLENK | 1212.6714 | AFD97756 |  |  | 100 |
| 13020 | LLESYMD**G**CVLGK | 1426.6836 | AFD97756 |  |  | 92 |
|  |  |  |  |  |  |  |
| 5328 | YQTSFK | 772.3755 | AFF18065 | BmorCSP2 | BABH01021427 | 100 |
| 5620 | LPEALETHC**G**AK | 1267.623 | AFF18063 |  |  | 92 |
| 9741 | SFDASEVLSNER | 1352.6208 | AFF18063 |  |  | 100 |
| 10430 | **ASPGGLE**GPC**G**TAELKK | 1613.8083 | AFF18063 |  |  | 53 |
| 10587 | DSFDASEVLWDGK | 1467.6519 | AFF18063 |  |  | 100 |
| 11765 | **EPCCW**SEVLSNER | 1550.6494 | AFF18063 |  |  | 62 |
| 12672 | **CAHF**SEVLSNER | 1390.6299 | AFF18063 |  |  | 67 |
| 12673 | **EYPLCQ**SFDASEVLSNER | 2085.9312 | AFF18063 |  |  | 67 |
| 13001 | **FHA**YEPLDDSFDASEVLSNER | 2440.0818 | AFF18063 |  |  | 86 |
| 13016 | **YA**D**AP**EPLD**GY**FDASEVLSNER | 2457.0972 | AFF18063 |  |  | 73 |
|  |  |  |  |  |  |  |
| 3912 | HSTEWEK | 915.4086 | NP_001037400 | BmorCSP3 | BGIBMGA004042 | 100 |
| 11203 | YENFDVEPLVT**DS**R | 1682.7788 | NP_001037400 |  |  | 86 |
|  |  |  |  |  |  |  |
| 4739 | GCLLDK**DR** | 918.4593 | AFF18087 | BmorCSP4 | BGIBMGA004047 | 75 |
|  |  |  |  |  |  |  |
| 3417 | AHLKD**CT**QTA**G**CAK | 1445.6755 | NP_001037065 | BmorCSP6 | BGIBMGA004046 | 79 |
| 5712 | YDPKDEFK | 1040.4814 | NP_001037065 |  |  | 100 |
| 5753 | QHEADYWE**ACT**K | 1479.6089 | NP_001037065 |  |  | 75 |
| 7517 | QH**QMG**YWEQMK | 1464.6279 | NP_001037065 |  |  | 73 |
| 8698 | KLLVPYLK | 972.6371 | NP_001037065 |  |  | 100 |
| 10752 | **VSA**YTDKYDNLDVDELLENR | 2371.1179 | NP_001037065 |  |  | 85 |
| 10792 | **EDPPG**DVDELLENR | 1596.7266 | NP_001037065 |  |  | 64 |
| 11723 | YTDKYDNLDVDELLENR | 2113.9802 | NP_001037065 |  |  | 100 |
| 11836 | **MF**NLDVDELLENR | 1606.7661 | NP_001037065 |  |  | 85 |
| 12688 | YDNLDVDELLENR | 1606.7473 | NP_001037065 |  |  | 100 |
| 13727 | **WMAVDVACLTDPG**YDNLDVDELLDQR | 2965.3472 | NP_001037065 |  |  | 50 |
|  |  |  |  |  |  |  |
| 10062 | RNG**FVF**TR | 995.5301 | NP_001091778 | BmorCSP7 | BGIBMGA004067 | 62 |
|  |  |  |  |  |  |  |
| 4613 | EALETEC**G**AK | 1049.47 | NP_001037063 | BmorCSP8 | BGIBMGA004066 | 90 |
| 5098 | RVLGHLLNNESK | 1378.7681 | NP_001037063 |  |  | 100 |
| 5699 | VLGHLLNNESK | 1222.667 | NP_001037063 |  |  | 100 |
| 6226 | VLGHLL**D**NESK | 1223.6509 | NP_001037063 |  |  | 91 |
| 6413 | VLGHLLN**D**ESK | 1223.6509 | NP_001037063 |  |  | 91 |
| 6570 | VLGHLL**D**NESK | 1223.6509 | NP_001037063 |  |  | 91 |
| 7387 | **LV**PDA**LSN**K | 955.5338 | NP_001037063 |  |  | 44 |
| 7650 | SWNELTAK | 947.4713 | NP_001037063 |  |  | 100 |
| 10733 | **NVSYWDY**VNLDEVLSNSR | 2172.0122 | NP_001037063 |  |  | 61 |
| 10810 | **QPDSN**NLDEVLSNSR | 1686.7808 | NP_001037063 |  |  | 67 |
| 11544 | YDNVNLDEVLSNSR | 1636.7693 | NP_001037063 |  |  | 100 |
|  |  |  |  |  |  |  |
| 3899 | KYETELKR | 1065.5818 | NP_001037062 | BmorCSP9 | BGIBMGA004065 | 100 |
| 4314 | KYETELK | 909.4807 | NP_001037062 |  |  | 100 |
| 6183 | YDPTNEFTKK | 1241.5928 | NP_001037062 |  |  | 100 |
| 6932 | YDPT**D**EFTK | 1114.4819 | NP_001037062 |  |  | 89 |
| 6293 | AKYDPT**GG**EFTK | 1312.6299 | NP_001037062 |  |  | 83 |
| 7520 | YDPTNEFTK | 1113.4978 | NP_001037062 |  |  | 100 |
| 8187 | YDPT**D**EFTK | 1114.4819 | NP_001037062 |  |  | 89 |
| 10718 | EQYTDKYDTVD**EVWPKAKT**R | 2471.1968 | NP_001037062 |  |  | 60 |
| 12107 | **QE**YTDKYDTVDLDQLLSNR | 2315.0916 | NP_001037062 |  |  | 89 |
| 13182 | YDTVDLDQLLSNR | 1550.7576 | NP_001037062 |  |  | 100 |
|  |  |  |  |  |  |  |
| 5508 | RLPEALR | 853.5134 | NP_001037067 | BmorCSP11 | BGIBMGA004040 | 100 |
| 8518 | **CPD**TAEGTEFKK | 1324.5969 | NP_001037067 |  |  | 75 |
| 10298 | C**G**FLDQGPC**G**TAEGTEFK | 1858.7866 | NP_001037067 |  |  | 89 |
| 11182 | HEFTAF**CTDNK** | 1311.5554 | NP_001037067 |  |  | 55 |
| 11650 | **EHH**DQGPC**G**TAEGTEFKK | 1969.8589 | NP_001037067 |  |  | 78 |
| 13392 | LPDLWEELALKEDPK | 1794.9402 | NP_001037067 |  |  | 100 |
| 14119 | NFDVEQLVGNLR | 1402.7205 | NP_001037067 |  |  | 100 |
| 14261 | FDVEQLVNGLR | 1288.6775 | NP_001037067 |  |  | 100 |
| 14623 | **HHE**DQGP**G**CTAEGTEFK | 1841.7639 | NP_001037067 |  |  | 71 |
| 14643 | LPDL**SMT**ELAL**EDK** | 1573.7908 | NP_001037067 |  |  | 64 |
| 15291 | LPDLWEELALK | 1325.7231 | NP_001037067 |  |  | 100 |
| 15310 | **YSGD**YSS**GA**YDNFDVEQLVGNLR | 2568.1404 | NP_001037067 |  |  | 65 |
| 15830 | EEY**S**SS**C**EQLVGNLR | 1712.7676 | NP_001037067 |  |  | 87 |
| 15524 | LPDLWEELAL**DEK** | 1569.7925 | NP_001037067 |  |  | 77 |
| 17337 | **CSEC**FDVEQLVGNLR | 1710.7705 | NP_001037067 |  |  | 73 |
| 17346 | CHYYSSQYDNFDVEQLVGNLR | 2549.1279 | NP_001037067 |  |  | 90 |
|  |  |  |  |  |  |  |
| 8026 | **SR**LPQEYEAFK | 1366.688 | NP_001037068 | BmorCSP12 | BGIBMGA004041 | 82 |
| 8849 | DLPEAVAEAC**G**GK | 1258.5862 | NP_001037068 |  |  | 100 |
| 14683 | **EKSSSC**EALVGNLDSLK | 1778.8721 | NP_001037068 |  |  | 65 |
|  |  |  |  |  |  |  |
| 4114 | YKDKLDAVK | 1078.6023 | AFF18177 | BmorCSP14 | BGIBMGA004068 | 100 |
| 7153 | **DY**PDNLYQAR | 1253.5676 | AFF18122 |  |  | 80 |
| 7814 | YDPDNLYQAR | 1253.5676 | AFF18142 |  |  | 100 |
| 13168 | **TDHGP**NVDELLESNR | 1694.7859 | AFF18142 |  |  | 67 |
| 13700 | WDNLNVDELLESNR | 1715.8115 | AFF18142 |  |  | 100 |
|  |  |  |  |  |  |  |
| 7061 | **AA**YDDF**AK** | 899.4025 | NP_001091779 | BmorCSP15 | BGIBMGA004044 | 50 |
| 8196 | L**V**PEALETTC**G**GK | 1316.6646 | [NP_001091779](http://www.ncbi.nlm.nih.gov/protein/148298882?report=genbank&log$=protalign&blast_rank=2&RID=833ZX87N01R) |  |  | 85 |
| 8903 | HPEAWEELVNK | 1350.6567 | NP_001091779 |  |  | 100 |
|  |  |  |  |  |  |  |
| 4534 | LT**E**EQKAELTK | 1288.6875 | NP_001140190 | BmorOBP6 | BGIBMGA008354 | 91 |
| 4602 | LTET**G**CA**EP**NDKPVDSDSK**D**C**Q**R | 2507.0903 | NP_001140190 |  |  | 78 |
| 5790 | KYLTSEEDLK | 1224.6238 | NP_001140190 |  |  | 100 |
| 6915 | YLTSEEDLK | 1096.5288 | NP_001140190 |  |  | 100 |
| 6921 | EFSVNQGDLDAAKK | 1520.7471 | NP_001140190 |  |  | 100 |
| 8276 | EFSVN**GA**GDLDAAK | 1392.6521 | NP_001140190 |  |  | 86 |
| 9095 | YLTSEEDLKAFEK | 1571.7717 | NP_001140190 |  |  | 100 |
| 9395 | SSLPLLAEC**G**SK | 1203.6169 | NP_001140190 |  |  | 92 |
| 10371 | SP**G**LNSC**G**FVGC**G**M**Y**K | 1618.6941 | NP_001140190 |  |  | 75 |
| 12458 | LLLDC**G**FVANK | 1191.6321 | NP_001140190 |  |  | 91 |
| 15327 | L**Q**LNASGLFDVAATLEK | 1788.9622 | NP_001140190 |  |  | 94 |
| 16573 | **L**G**A**L**D**ASGLFDVAATLEK | 1789.946 | NP_001140190 |  |  | 83 |
|  |  |  |  |  |  |  |
| 9279 | LLSQVAAASFPK | 1230.6973 | NP_001153665 | OBP | BGIBMGA002629 | 100 |
| 9402 | LVSFAPEVAK | 1059.5964 | NP_001153665 |  |  | 100 |
| 11495 | A**AD**YREDEPFQNLVYC**G**AYK | 2351.0527 | NP_001153665 |  |  | 85 |
| 13711 | LD**D**VVTVLESC**G**GK | 1433.7073 | NP_001153665 |  |  | 86 |
| 14148 | **HPAD**GLGEDALEVLR | 1590.8 | NP_001153665 |  |  | 73 |
|  |  |  |  |  |  |  |
| 4628 | ADEQLVNK | 915.4661 | NP_001040212 | Sericotropin | BGIBMGA010010 | 100 |
|  |  |  |  |  |  |  |
| 4795 | LPPGVNK | 723.4279 | XP_004932433 | GOBP56d | BGIBMGA002666 | 100 |
| 5397 | FAACTLEK | 881.4316 |  |  |  |  |
|  |  |  |  |  |  |  |
| 8712 | VKE**G**CLELFPK | 1261.6741 | XP_004933865 | PBPRP3 | BGIBMGA002630 | 91 |
|  |  |  |  |  |  |  |
| 7535 | T**D**GVSTEVLNAAK | 1303.6619 | XP_004932341 | B1 | BGIBMGA002627 | 92 |
|  |  |  |  |  |  |  |
| ***Lys-C*** |  |  |  |  |  |  |
| 4604 | LNLQELLENK | 1212.6714 | AFD97756 | BmorCSP1 | BGIBMGA004045 | 100 |
|  |  |  |  |  |  |  |
| 1935 | QLAQGLKK | 884.5443 | AFF18063 | BmorCSP2 | BABH01021427 | 100 |
| 2282 | QLAQGLK | 756.4493 | AFF18063 |  |  | 100 |
| 2534 | YQTSFK | 772.3755 | AFF18065 |  |  | 100 |
| 2654 | LPEALETH**Q**CK | 1267.623 | AFF18063 |  |  | 91 |
| 4815 | Q**PE**YEPLDDSFDASEVLSNERLLK | 2793.3342 | AFF18063 |  |  | 92 |
| 5396 | TH**EP**LWDEFLTFYDPQGK | 2222.032 | AFF18065 |  |  | 89 |
|  |  |  |  |  |  |  |
| 3227 | VLRHL**LDNK**P**EM**W**A**K | 1849.0032 | AFF18115 | BmorCSP14 | BGIBMGA004068 | 60 |
|  |  |  |  |  |  |  |
| 4674 | LL**T**NDRL**F**L**N**YFK | 1655.9036 | NP_001037192 | BmorCSP17 | BGIBMGA004043 | 77 |
|  |  |  |  |  |  |  |
| 2589 | TAEV**NPGC**VTAHFGK | 1529.7297 | NP_001037498 | BmorGOBP2 | BGIBMGA012614 | 73 |
| 3177 | TAEVMSHVT**HA**FGK | 1513.7346 | NP_001037498 |  |  | 86 |
| 3118 | TAEVMSHVTAHFGK | 1513.7346 | NP_001037498 |  |  | 100 |
| 3466 | **NSG**DTETDD**G**CTRVVK | 1695.7371 | NP_001037498 |  |  | 75 |
| 5045 | **L**G**E**APEV**S**M**V**EAVLEK | 1699.8701 | NP_001037498 |  |  | 75 |
|  |  |  |  |  |  |  |
| 4518 | LLLDC**G**FVANK | 1191.6321 | NP_001140190 | BmorOBP6 | BGIBMGA008354 | 91 |
