# Supplementary material for: Molecular Evidence of RNA Editing in Bombyx Chemosensory Protein Family
Source: PLoS One. 2014 Feb 13;9(2):e86932. doi: 10.1371/journal.pone.0086932 (PMC3923736; doi:10.1371/journal.pone.0086932)
Supplement: Table S4 — P2 de novo peptide sequences. Amino acid replacements are shown in bold and underlined. (DOC) [file pone.0086932.s011.doc]

| **Peptide No.** | **Peptide Sequence** | **Peptide Mass (Da)** | **NCBI (Blastp)** | **Protein Name** | **Gene Acc. No.** | **Identity (%)** |
| --- | --- | --- | --- | --- | --- | --- |
| ***Trypsin*** |  |  |  |  |  |  |
| 3977 | DDKYTDKYDK | 1289.5774 | AFD97756 | BmorCSP1 | BGIBMGA004045 | 100 |
| 6622 | DHLQEALETG**G**CEK | 1528.6826 | AFF18017 |  |  | 93 |
| 6651 | L**DKK**KELTAHFDPDGK | 1840.9683 | AFF18017 |  |  | 81 |
| 6656 | DHLQEALETGC**G**EK | 1528.6826 | AFF18017 |  |  | 93 |
| 6663 | ELTAHFDPDGK | 1228.5723 | AFF18017 |  |  | 100 |
| 10577 | LNLQELLENKR | 1368.7725 | AFD97756 |  |  | 100 |
| 12825 | LNLQELLENK | 1212.6714 | AFD97756 |  |  | 100 |
| 13282 | LLESYMD**G**CVLGK | 1426.6836 | AFD97756 |  |  | 93 |
|  |  |  |  |  |  |  |
| 5431 | LPEALETH**G**CAK | 1267.623 | AFF18063 | BmorCSP2 |  | 92 |
| 8661 | **VA**CL**NGA**GPC**G**TAELK | 1502.7222 | AFF18063 |  |  | 6 3 |
| 10566 | DSFDASEVLSNER | 1467.6477 | AFF18063 |  |  | 100 |
| 12887 | YEPLDDSFDASEVLSNER | 2084.9172 | AFF18063 |  |  | 100 |
| 13176 | **EQP**YELPDDSFDASEVLSENR | 2439.0713 | AFF18063 |  |  | 86 |
| 13273 | **EPQ**YEPLDDSFDASEVLSNER | 2439.0713 | AFF18063 |  |  | 86 |
| 13284 | **YA**D**PA**EPLDD**GPP**DASEVLSNER | 2456.0979 | AFF18063 |  |  | 70 |
|  |  |  |  |  |  |  |
| 6868 | **EDP**EASDFKK | 1164.5298 | NP_001037400 | BmorCSP3 | BGIBMGA004042 | 70 |
| 7099 | **LA**PDTLATNC**G**GK | 1259.6179 | NP_001037400 |  |  | 81 |
| 10390 | AYLN**G**CFLDK | 1142.543 | NP_001037400 |  |  | 90 |
| 11247 | YENFDVEPLVTSDR | 1682.7788 | NP_001037400 |  |  | 100 |
|  |  |  |  |  |  |  |
| 4655 | GCLLDK**DR** | 918.4593 | AFF18087 | BmorCSP4 | BGIBMGA004047 | 75 |
| 6781 | Y**F**E**SQK**K | 928.4654 | AFF18079 |  |  | 43 |
|  |  |  |  |  |  |  |
| 5436 | QHEA**A**YWE**CAT**K | 1435.6191 | NP_001037065 | BmorCSP6 | BGIBMGA004046 | 67 |
| 5516 | YDPKDEFK | 1040.4814 | NP_001037065 |  |  | 100 |
| 5547 | QHEADYWE**CATK** | 1479.6089 | NP_001037065 |  |  | 67 |
| 5648 | YDPKDEFK | 1040.4814 | NP_001037065 |  |  | 100 |
| 5677 | QHEADYWE**ACTK** | 1479.6089 | NP_001037065 |  |  | 67 |
| 5703 | Y**G**DPKDEFK | 1097.5029 | NP_001037065 |  |  | 89 |
| 6808 | VDELLENR | 986.5032 | NP_001037065 |  |  | 100 |
| 7248 | QHEADY**RCG**QMK | 1464.6238 | NP_001037065 |  |  | 75 |
| 8497 | KLLVPYLK | 972.6371 | NP_001037065 |  |  | 100 |
| 8650 | KLLVPYLK | 972.6371 | NP_001037065 |  |  | 100 |
| 9012 | **HWVG**DVDELLENR | 1580.7583 | NP_001037065 |  |  | 69 |
| 9403 | Q**CF**ADYWEQMK | 1447.5901 | NP_001037065 |  |  | 82 |
| 9339 | **NSQDCCCC**HEADYW**QE**MK | 2191.7524 | NP_001037065 |  |  | 56 |
| 10237 | YTDKYDNLDVDELLE**K**NR | 2242.0752 | NP_001037065 |  |  | 94 |
| 10330 | ELYEGFLAG**GAGK** | 1310.6506 | NP_001037065 |  |  | 69 |
| 10772 | **EDPGP**DVDELLENR | 1596.7266 | NP_001037065 |  |  | 64 |
| 10774 | **TVG**YTDKYDNLDVDELLENR | 2371.1179 | NP_001037065 |  |  | 89 |
| 10780 | **TNMY**DVDELLENR | 1610.7246 | NP_001037065 |  |  | 69 |
| 10788 | **THCCA**DVDELLENR | 1616.6924 | NP_001037065 |  |  | 64 |
| 10833 | **QVS**Y**ART**YDNLDVDELLENR | 2412.1555 | NP_001037065 |  |  | 70 |
| 10887 | ELYEGFLAGQ**DEK** | 1497.6987 | NP_001037065 |  |  | 77 |
| 10893 | **H**DNLDVDELLENR | 1580.7429 | NP_001037065 |  |  | 92 |
| 10993 | **SLG**Y**ES**KYDNLDVEELLENR | 2385.1335 | NP_001037065 |  |  | 75 |
| 11060 | **TVG**YTDKYDNLDVDELLE**D**R | 2372.1018 | NP_001037065 |  |  | 80 |
| 11816 | YTDKYDNLDVDELLENR | 2113.9802 | NP_001037065 |  |  | 100 |
| 11834 | YTDKYD**GG**LDVDELLENR | 2113.9802 | NP_001037065 |  |  | 89 |
| 12003 | YTDKYDNLDVDELLENR | 2113.9802 | NP_001037065 |  |  | 100 |
| 12166 | YTDKYDNLDVDELLENR | 2113.9802 | NP_001037065 |  |  | 100 |
| 13130 | YDNLDVDELLENR | 1606.7473 | NP_001037065 |  |  | 100 |
| 13168 | YDN**YS**VDELLENR | 1628.7317 | NP_001037065 |  |  | 85 |
| 11636 | **EDCDT**DVDELLENR | 1664.6836 | NP_001037065 |  |  | 64 |
| 11816 | YTDKYDNLDVDELLENR | 2113.9802 | NP_001037065 |  |  | 100 |
| 11939 | **MF**NLDVDELLENR | 1606.7661 | NP_001037065 |  |  | 85 |
| 12166 | YTDKYDNLDVDELLENR | 2113.9802 | NP_001037065 |  |  | 100 |
| 12340 | **TVGMS**DVDELLENR | 1576.7402 | NP_001037065 |  |  | 64 |
| 12354 | **Q**VDVDELLENR | 1328.6572 | NP_001037065 |  |  | 91 |
| 12424 | LDVDELLENR | 1214.6143 | NP_001037065 |  |  | 100 |
| 12500 | **TQYS**DVDELLENR | 1580.7317 | NP_001037065 |  |  | 69 |
| 13399 | YDNLDVDELLENR | 1606.7473 | NP_001037065 |  |  | 100 |
| 13718 | ELYEGFLAG**MK** | 1256.6111 | NP_001037065 |  |  | 82 |
| 14081 | **TPDG**YDNLDVDELLENR | 1976.8962 | NP_001037065 |  |  | 77 |
| 16002 | **SVGCA**YDNLDVDELLENR | 2023.9155 | NP_001037065 |  |  | 72 |
|  |  |  |  |  |  |  |
| 4549 | EALETEC**G**AK | 1049.47 | NP_001037063 | BmorCSP8 | BGIBMGA004066 | 90 |
| 4954 | RVLGHLLNNESK | 1378.7681 | NP_001037063 |  |  | 100 |
| 5498 | VLGHLLNNESK | 1222.667 | NP_001037063 |  |  | 100 |
| 6157 | VLGHLLN**D**ESK | 1223.6509 | NP_001037063 |  |  | 91 |
| 6320 | VLGHLL**D**NESK | 1223.6509 | NP_001037063 |  |  | 91 |
| 6467 | AL**SV**E**ED**CAK | 1063.4856 | NP_001037063 |  |  | 60 |
| 10714 | YTDR**DY**NVNLDEVLSNSR | 2172.0083 | NP_001037063 |  |  | 89 |
| 10818 | **DDPD**VNLDEVLSNSR | 1686.7695 | NP_001037063 |  |  | 73 |
| 11113 | **STGNYSGF**NVNLDEVLSDSR | 2172.9922 | NP_001037063 |  |  | 60 |
| 11632 | YDNVNLDEVLSNSR | 1636.7693 | NP_001037063 |  |  | 100 |
|  |  |  |  |  |  |  |
| 4284 | KYETELK | 909.4807 | NP_001037062 | BmorCSP9 | BGIBMGA004065 | 100 |
| 5948 | YDPTNEFTKK | 1241.5928 | NP_001037062 |  |  | 100 |
| 6044 | **KA**YDTPNEFTK | 1312.6299 | NP_001037062 |  |  | 82 |
| 7262 | YDPTNEFTK | 1113.4978 | NP_001037062 |  |  | 100 |
| 7416 | YDPTNEFTK | 1113.4978 | NP_001037062 |  |  | 100 |
| 10970 | LLNHEAEFWEELK | 1656.8147 | NP_001037062 |  |  | 100 |
| 11306 | YDTVDLDQL**GKRE**R | 1706.8586 | NP_001037062 |  |  | 71 |
| 11654 | **LNM**AEFWEELK | 1408.6697 | NP_001037062 |  |  | 73 |
| 12266 | **QE**YTDKYDTVDLDQLLSNR | 2315.0916 | NP_001037062 |  |  | 89 |
| 12303 | **EQQH**DKYDTVDLDQLLSNR | 2316.0981 | NP_001037062 |  |  | 74 |
| 13476 | YDTVDLDQLLSNR | 1550.7576 | NP_001037062 |  |  | 100 |
| 14092 | **DTW**TDKYDTVDLDQLLSNR | 2297.0811 | NP_001037062 |  |  | 84 |
|  |  |  |  |  |  |  |
| 7750 | VEQLVGNLR | 1026.5822 | NP_001037067 | BmorCSP11 | BGIBMGA004040 | 100 |
| 8320 | **CPD**TAEGTEFKK | 1324.5969 | NP_001037067 |  |  | 75 |
| 10247 | **GC**FLDQGP**G**CTAEGTEFK | 1858.7866 | NP_001037067 |  |  | 89 |
| 10552 | HEFTAF**MDAK** | 1195.5332 | NP_001037067 |  |  | 60 |
| 11072 | HEFTAF**QCSK** | 1196.5283 | NP_001037067 |  |  | 60 |
| 11128 | VEQ**D**LV**F**LR | 1117.613 | NP_001037067 |  |  | 77 |
| 11737 | **GQSM**DQGPC**G**TAEGTEFKK | 1969.8508 | NP_001037067 |  |  | 74 |
| 11763 | **WMS**DQGPC**G**TAEGTEFKK | 1970.8501 | NP_001037067 |  |  | 78 |
| 12049 | **NKS**D**CG**GPCTAEGTEFK | 1742.7239 | NP_001037067 |  |  | 71 |
| 13701 | LPDLWEELALKEDPK | 1794.9402 | NP_001037067 |  |  | 100 |
| 14559 | NFDVEQLVGNLR | 1402.7205 | NP_001037067 |  |  | 100 |
| 14724 | FDVEQLVGNLR | 1288.6775 | NP_001037067 |  |  | 100 |
| 15154 | **EHH**DQGP**G**CTAEGTEFK | 1841.7639 | NP_001037067 |  |  | 76 |
| 15913 | LPDLWEELALK | 1325.7231 | NP_001037067 |  |  | 100 |
| 15928 | **GSD**YYSSQYDNFDVEQLVGNLR | 2568.1404 | NP_001037067 |  |  | 86 |
| 16074 | LPDLWEELALK | 1325.7231 | NP_001037067 |  |  | 100 |
| 16177 | LPDLWEELAL**DEK** | 1569.7925 | NP_001037067 |  |  | 77 |
| 18240 | **YSDSN**SSQYDNFDVEQLVGNLR | 2549.1306 | NP_001037067 |  |  | 72 |
| 18247 | **MMGC**FDVEQLVGNLR | 1710.7891 | NP_001037067 |  |  | 73 |
|  |  |  |  |  |  |  |
| 5787 | GCF**Q**ETSP**VR** | 1122.5127 | NP_001037068 | BmorCSP12 | BGIBMGA004041 | 70 |
| 6574 | FLEVVKDK | 976.5593 | NP_001037068 |  |  | 100 |
| 7768 | **SR**LPQEYEAFK | 1366.688 | NP_001037068 |  |  | 82 |
| 8100 | FLEVVK | 733.4374 | NP_001037068 |  |  | 100 |
| 8673 | LDPEAVAEAC**G**GK | 1258.5862 | NP_001037068 |  |  | 92 |
| 10842 | FLEVVKDKLP**AG**EYEAFK | 2082.1035 | NP_001037068 |  |  | 90 |
| 16719 | LDLEALVGNLDSLK | 1498.8242 | NP_001037068 |  |  | 100 |
|  |  |  |  |  |  |  |
| 4101 | YKDKLDAVK | 1078.6023 | AFF18177 | BmorCSP14 | BGIBMGA004068 | 100 |
| 5971 | RPDLWK | 813.4497 | AFF18177 |  |  | 100 |
| 7562 | YDPDNLYQAR | 1253.5676 | AFF18177 |  |  | 100 |
| 8403 | ETLPDALEHE**G**CVK | 1539.7239 | AFF18137 |  |  | 93 |
| 11719 | **QV**NVDELLESNR | 1414.7051 | AFF18142 |  |  | 83 |
| 13459 | **TSMNFL**D**TT**DNLNVDELLESNR | 2540.1699 | AGR44916 |  |  | 62 |
| 14058 | WDNLNVDELLE**NS**R | 1715.8115 | AFF18142 |  |  | 86 |
|  |  |  |  |  |  |  |
| 5200 | EFYSSR | 787.35 | NP_001091779 | BmorCSP15 | BGIBMGA004044 | 100 |
| 7945 | **L**VPEALETTC**G**GK | 1316.6646 | NP_001091779 |  |  | 85 |
| 8730 | HPEAWEELVNK | 1350.6567 | NP_001091779 |  |  | 100 |
| 9644 | YDDFDVKPLVENDR | 1723.8052 | NP_001091779 |  |  | 100 |
| 10565 | LLQSYTN**G**CFLDK | 1500.7283 | NP_001091779 |  |  | 92 |
|  |  |  |  |  |  |  |
| 5589 | KYLTSEEDLK | 1224.6238 | NP_001140190 | BmorOBP6 | BGIBMGA008354 | 100 |
| 6650 | **FE**SVNQGDLDAAKK | 1520.7471 | NP_001140190 |  |  | 86 |
| 6655 | YLTSEEDLK | 1096.5288 | NP_001140190 |  |  | 100 |
| 6799 | YLTSEEDLK | 1096.5288 | NP_001140190 |  |  | 100 |
| 8058 | EFSVNQGDLDAAK | 1392.6521 | NP_001140190 |  |  | 100 |
| 8936 | YLTS**GSN**DLKAFEK | 1571.7832 | NP_001140190 |  |  | 79 |
| 11568 | A**KD**Y**NN**DEPFQNLVYC**G**AYK | 2351.0527 | NP_001153665 |  |  | 75 |
| 12662 | LLLDC**G**FVANK | 1191.6321 | NP_001140190 |  |  | 91 |
| 15949 | **L**G**A**LNASGLFDVAATLEK | 1788.9622 | NP_001140190 |  |  | 89 |
| 17394 | **VRVD**ASGLFDVAATLEK | 1789.9573 | NP_001140190 |  |  | 76 |
|  |  |  |  |  |  |  |
| 7749 | KLVSFAPEVAK | 1187.6914 | NP_001153665 | OBP | BGIBMGA002629 | 100 |
| 9131 | LLSQVAAASFPK | 1230.6973 | NP_001153665 |  |  | 100 |
| 14079 | L**D**DVVTVLESC**G**GK | 1433.7073 | NP_001153665 |  |  | 86 |
| 14609 | **LV**LQEC**G**LNE**D**GLGEDALEVLR | 2384.1892 | NP_001153665 |  |  | 82 |
|  |  |  |  |  |  |  |
| 2601 | NGSPHQTAWNYVK | 1500.7109 | NP_001040212 | Sericotropin | BGIBMGA010010 |  |
| 6267 | KNFCQTAWNYVK | 1500.7183 |  |  |  |  |
|  |  |  |  |  |  |  |
| 5787 | **G**CFQETSPVR | 1122.5127 | XP_004933865 | PBPRP3 | BGIBMGA002630 | 90 |
| 8525 | VKE**G**CLELFPK | 1261.6741 | XP_004933865 |  |  | 91 |
|  |  |  |  |  |  |  |
| 6111 | SEAQSVLEQC**G**K | 1277.592 | XP_004932341 | B1 | BGIBMGA002627 | 92 |
|  |  |  |  |  |  |  |
| ***Lys-C*** |  |  |  |  |  |  |
| 579 | KYEDRAK | 908.4716 | AFF18032 | BmorCSP1 | BGIBMGA004045 | 100 |
| 2568 | ELTAHFDPDGK | 1228.5723 | AFF18017 |  |  | 100 |
| 2661 | ELTAHFDPDGK | 1228.5723 | AFF18017 |  |  | 100 |
| 2662 | ELTAHFDPDGK | 1228.5723 | AFF18017 |  |  | 100 |
| 2741 | DHLQEALETG**ECGK** | 1528.6826 | AFF18017 |  |  | 71 |
| 3413 | NELELWK | 930.4811 | AFF18017 |  |  | 100 |
| 3543 | **Q**ETSLDYLLK | 1208.6289 | AFF18017 |  |  | 90 |
| 4035 | LNLQELLENK | 1212.6714 | AFD97756 |  |  | 100 |
|  |  |  |  |  |  |  |
| 2123 | QLA**E**GLK | 757.4333 | AFF18063 | BmorCSP2 |  | 86 |
| 2187 | YQTSFK | 772.3755 | AFF18065 |  |  | 100 |
| 2248 | FYDPQGK | 853.397 | AFF18065 |  |  | 100 |
| 2301 | LPEALETHC**Q**K | 1267.623 | AFF18063 |  |  | 91 |
| 2302 | LPEALETHC**G**AK | 1267.623 | AFF18063 |  |  | 92 |
| 2397 | **KD**LPEALETHC**Q**K | 1510.7449 | AFF18063 |  |  | 77 |
| 2422 | LPEALETH**SNSD**TDK | 1655.7639 | AFF18056 |  |  | 73 |
| 3994 | YEPLDDSFDASEVL**GWDK** | 2084.9214 | AFF18063 |  |  | 78 |
|  |  |  |  |  |  |  |
| 2125 | GCLLDK | 647.3312 | AFF18087 | BmorCSP4 | BGIBMGA004047 | 100 |
|  |  |  |  |  |  |  |
| 2799 | SWNELTAK | 947.4713 | NP_001037063 | BmorCSP8 | BGIBMGA004066 | 100 |
|  |  |  |  |  |  |  |
| 1881 | KYETELK | 909.4807 | NP_001037062 | BmorCSP9 | BGIBMGA004065 | 100 |
|  |  |  |  |  |  |  |
| 2289 | GEYTENLDK | 1067.4771 | NP_001037066 | BmorCSP13 | BGIBMGA004035 | 100 |
| 2314 | LHDPKGEYTENLDK | 1657.7947 | NP_001037066 |  |  | 100 |
| 3045 | NDDLFYDK | 1028.4451 | NP_001037066 |  |  | 100 |
| 3099 | D**FQ**PDEFDLLRK | 1521.7463 | NP_001037066 |  |  | 83 |
| 3100 | **TVCS**PDEFDLLRK | 1521.7498 | NP_001037066 |  |  | 69 |
| 3161 | DDLFYDK | 914.4021 | NP_001037066 |  |  | 100 |
| 3357 | AYTF**G**CFNDK | 1164.491 | NP_001037066 |  |  | 90 |
| 3507 | AYTF**G**CF**D**DK | 1165.4749 | NP_001037066 |  |  | 80 |
|  |  |  |  |  |  |  |
| 2601 | N**G**SPHQTAWNYVK | 1500.7109 | NP_001040212 | Sericotropin | BGIBMGA010010 | 92 |
